# A Single Nucleotide Polymorphism within the *Interferon Gamma Receptor 2* Gene Perfectly Coincides with Polledness in Holstein Cattle

**DOI:** 10.1371/journal.pone.0067992

**Published:** 2013-06-21

**Authors:** Sabrina Glatzer, Nina Johanna Merten, Claudia Dierks, Anne Wöhlke, Ute Philipp, Ottmar Distl

**Affiliations:** Institute for Animal Breeding and Genetics, University of Veterinary Medicine Hannover, Hannover, Germany; University of Sydney, Australia

## Abstract

Polledness is a high impact trait in modern milk and beef production to meet the demands of animal welfare and work safety. Previous studies have mapped the polled-locus to the proximal region of the bovine chromosome 1 (BTA1) and narrowed it down to approximately 1 Mb. Sequencing of the positional candidate genes within the 1 Mb polled region and whole genome sequencing of Holsteins revealed a single nucleotide polymorphism (SNP) *AC000158: g.1390292G>A* within intron 3 of the *interferon gamma* receptor *2* gene (*IFNGR2*) in perfect co-segregation with polledness in Holsteins. This complete association was validated in 443 animals of the same breed. This SNP allows reliable genotyping of horned, heterozygous and homozygous polled Holsteins, even in animals that could not be resolved using the previously published haplotype for Holstein.

## Introduction

Breeding genetically polled cattle offers many advantages for modern beef and milk production. Polled animals are usually easier to handle, safer to work with and are less aggressive among each other. The risk of injuries causing depression in milk production, insufficiency in beef-quality and faults in leather are decreased. In Germany, dehorning of calves without anaesthetics is allowed until the age of 6 weeks. Nevertheless, this procedure is connected with pain for the animal and can lead to extensive damage if there are complications. Due to animal welfare affairs, a prohibition of dehorning in Germany is foreseeable. Furthermore, dehorning is rendered unnecessary when breeding genetically polled cattle and thus, the farmer can save time and money.

As well as rumination and cloven hooves, pneumatised horns are a typical characteristic of the bovine species. However, the appearance of polled cattle centuries ago is proven by prehistoric carvings in Scotland from before recorded history and Egyptian sculptures and paintings from around 3000 before Christ [1].

Polledness in cattle is an autosomal dominant trait [2–4]. The polled-locus was mapped to the centromeric region of BTA1 [5–8] and narrowed down between the microsatellites *RP42-218J17_MS1* (661 kb) and *BM6438* (2,025 kb) [9]. This area shows conserved synteny with human chromosome 21 (HSA1) [10,11].

A window of nine SNPs from 488 kb to 1,338 kb on BTA1 showed the greatest composite log likelihood (CLL) for the polled condition comparing a subset holding significant numbers of polled individuals to the breeds in the International Bovine HapMap study (IBHM). The SNP featuring the largest difference in allelic frequency between polled and horned animals was located within the *interferon gamma* receptor *2* gene (*IFNGR2*) [12].

Genotyping 285 animals of different cattle breeds with the bovine SNP50 BeadChip (Illumina) displayed a region of 381 kb on BTA1 where all 101 polled animals were homozygous for all genotyped SNPs [13]. This region harbours the three genes *histone* cluster *1, H4i like* (*HIST1H4C*)*, oligodendrocyte transcription* factor *1* (*OLIG1*) and *chromosome 1 open reading frame, human C21orf62* (*C1H21orf62*) and the two pseudogenes *oligodendrocyte transcription* factor *2 like* (*OLIG2 like*) and *40S ribosomal protein S13 like* (*LOC782947*). Using the Agilent 44k bovine array, expression levels from tissue-samples of the horn-forming region of polled and horned animals were examined. Differences in relative expression levels among horned and polled cattle could not be found for the genes located in the 1-Mb-polled-region on BTA1 [14].

A 202-bp-indel located between the genes *IFNAR2* and *OLIG1* is highly associated with polledness in several European cattle breeds, but not in Holsteins [15]. For Holsteins only a haplotype of seven intergenic polymorphisms was detected spanning an interval of 260 kb including the genes *HIST1H4C*, *OLIG1* and *C1H21orf62* and the pseudogenes *OLIG2 like* and *LOC782947* [15]. To our knowledge, no reliable single polymorphism differentiating polled and horned Holsteins has yet been found.

In this study, we screened the genes *mitochondrial ribosomal protein S6* (*MRPS6*), *solute carrier* family *5* (*sodium/myo-inositol cotransporter*)*,* member *3* (*SLC5A3*), *ATP synthase, H+ transporting, mitochondrial F1 complex, O subunit* (*ATP5O*), intersectin *1* (*SH3 domain protein*) (*ITSN1*), *crystallin, zeta* (*quinone reductase*)*-like 1* (*CRYZL1*), *downstream neighbor of SON* (*DONSON*), *SON DNA binding protein* (*SON*), *phosphoribosylglycinamide formyltransferase, phosphoribosylglycinamide synthetase, phosphoribosylaminoimidazole synthetase* (*GART*), *uncharacterized LOC784171*, *transmembrane* protein *50B* (*TMEM50B*), *interferon gamma* receptor *2* (*IFNGR2*), *interferon* (*alpha, beta and omega*) receptor *1* (*IFNAR1*), *interleukin 10 receptor, beta* (*IL10RB*), *interferon* (*alpha, beta and omega*) receptor *2* (*IFNAR2*), *HIST1H4C* and *OLIG1* and the pseudogenes *LOC782947* and *OLIG2-like* within the 1 Mb polled region for polymorphisms with high association to polledness in Holsteins and detected a SNP within the gene *interferon gamma* receptor *2* (*IFNGR2*) in complete co-segregation with polledness in 443 Holsteins.

## Results

### Mutation analysis and genotyping

**Figure 1:**
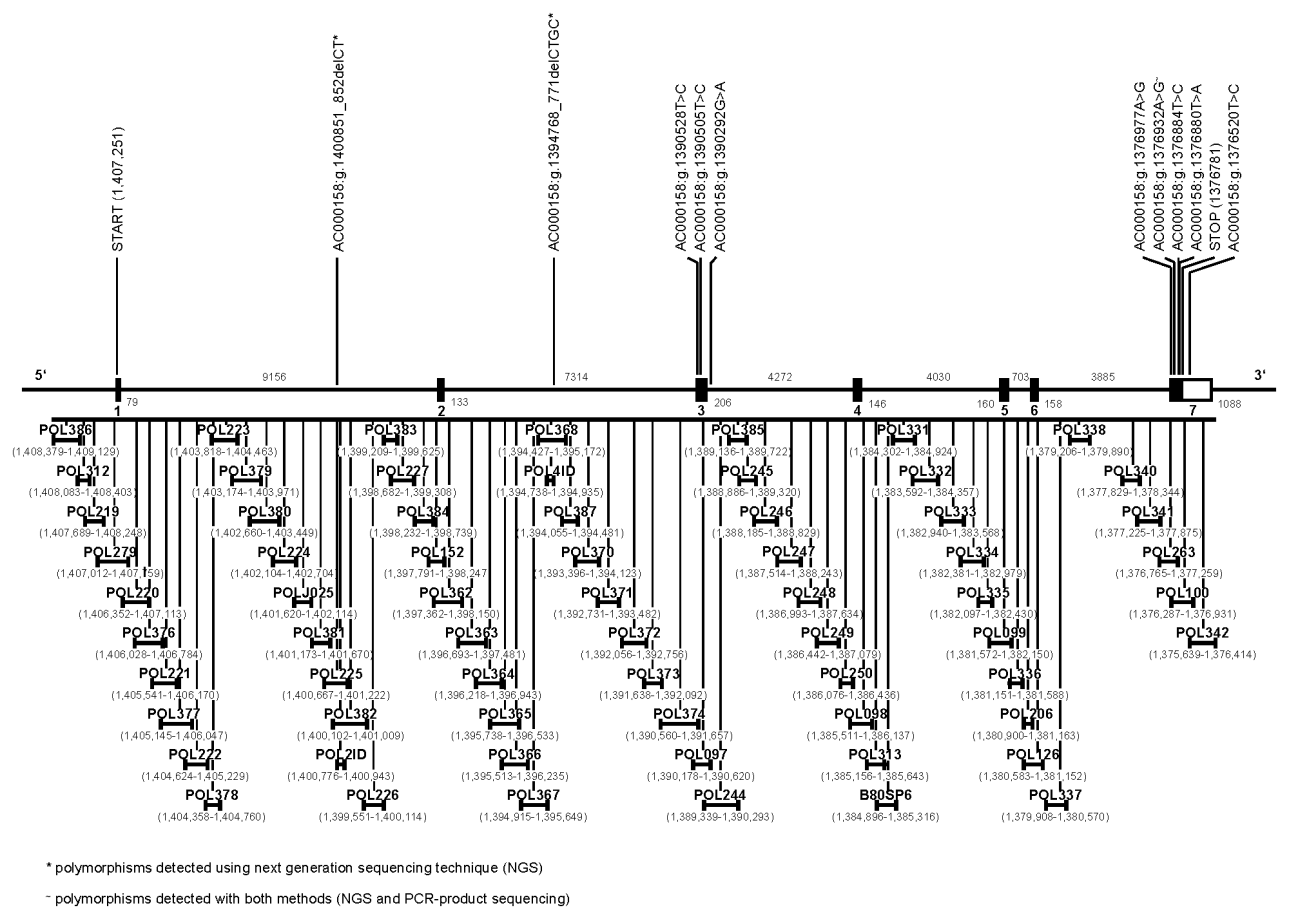
Gene structure of *IFNGR2*. Rectangles indicate exons. Coding sequence is filled with black, the 3’ UTR is filled with white. The size of exons and introns is specified in number of base pairs (bp). The black line below illustrates the sections analysed by PCR in Holsteins. Each PCR-product is pictured separately with start and end position. Position of start and stop codon is stated in bp. All detected exonic polymorphisms and intronic polymorphisms showing association with polledness in Holsteins are given with name and position above the gene model. Each position is given with accordance to Bos taurus assembly UMD3.1.

Highest association with polledness in Holsteins was identified for polymorphisms within the *interferon gamma* receptor *2* gene (*IFNGR2*). In total, 41 SNPs and two indel mutations were detected within *IFNGR2* by sequencing of PCR products (Table S1 Figure S1). Using next generation sequencing technique, further 18 SNPs and five indels were discovered within *IFNGR2* (Table S2 Figure S1). The SNPs *AC000158: g.1390505T>C*, *AC000158: g.1390528T>C, AC000158: g.1376880T>A, AC000158: g.1376884T>C, AC000158: g.1376932A>G* and *AC000158: g.1376977A>G* were localised within the coding sequence of exon 3 and 7 of *IFNGR2*, respectively (Figure 1). *AC000158: g.1376520T>C* was located within the 3’ UTR (Figure 1). All other polymorphisms were intronic. The indels *AC000158: g.1394768_771delCTGC* and *AC000158: g.1400851_852delCT* were not observed in breeds other than Holstein. We genotyped all SNPs and indels with high association to polledness in further 11 homozygous polled, 61 heterozygous polled and 336 horned Holsteins. Although most Holsteins were born in Germany or male animals had a German herdbook number, their genetic origin was highly diverse (Figure S2), as crossing with animals from different countries is common in Holstein breeding to improve milk yield. The ancestry is known for 82 of the 86 polled animals. Five of these animals were female. Two of these female Holsteins were born in Germany and Spain, respectively, and one female Holstein was born in the United Kingdom. Of the male Holsteins, 57% were born in Germany, 14% in the United States of America, 14% in the Netherlands, 5% in Canada, 4% in the Czech Republic, 3% in the United Kingdom and 1% in Spain and Belgium, respectively (Figure S3). Of these animals, 87% have ancestors from other than their native country. Parents came from Germany, the United States of America, the Netherlands, Canada or France and grandparents from Germany, the United States of America, the Netherlands, Canada, Italy or Austria. This high diversity of ancestry assures representation of many important polled lineages including animals from Aggravation, Burket-Falls, Hickorymea, Dansire, Sandy-Valley, West Port Holsteins, Arron Doon Holsteins, Golden Oaks, Baldus Holsteins and Hollysprings.

Polymorphisms that showed association in all Holsteins were genotyped in further 21 homozygous polled, 43 heterozygous polled and 28 horned German Fleckvieh and five homozygous polled, nine heterozygous polled and eight horned Charolais.

### Association analysis

The SNP *AC000158: g.1390292G>A*, within intron 3 showed a highly significant association with the polled genotype (χ^²^=423, -log_10_P=91.85) (Table 1 Figure 1) and perfect co-segregation in all 443 Holsteins. All horned Holsteins showed the genotype G/G, all heterozygous polled Holsteins the genotype G/A, and all homozygous polled Holsteins the genotype A/A. However, association could not be confirmed for the tested animals of the breeds Charolais and German Fleckvieh, as they had the genotype G/G in either case, whether they were horned or polled.

**Table 1:** Polymorphisms within the *interferon gamma* receptor *2* (*IFNGR2*) gene on BTA1 with association to polledness in Holsteins and exonic mutations in *IFNGR2* which failed association tests for polledness.

Polymorphism-ID	χ² genotype	-log_10_P	Influence on amino acid chain and protein function	Influence on transcription factor binding site
AC000158:g.1376520T>C	0.01	0.04		Activator protein 1
				(AP-1) binding site disrupted
AC000158:g.1376880T>A	2.44	0.93	missense (T>S),	
			probably damaging	
AC000158:g.1376884T>C	2.44	0.93	synonymous	
AC000158:g.1376932A>G	2.44	0.93	synonymous	
AC000158:g.1376977A>G	1.73	0.73	synonymous	
AC000158:g.1390292G>A	424.85	92.26		
AC000158:g.1390505T>C	0.33	0.25	missense (R>G); benign	
AC000158:g.1390528T>C	0.00		missense (K>R); benign	
AC000158:g.1394768_771delCTGC	70.37	15.18		
AC000158:g.1400851_852delCT	56.22	12.21		Interferon consensus sequence
				binding protein (ICSBP)
				binding site disrupted

For exonic polymorphisms their influence on the amino acid sequence and the protein structure analysed with PolyPhen-2 is given. For intronic polymorphisms their influence on transcription factor binding sites analysed with AliBaba2.1 is specified.

There was one progeny-proven pure-bred heterozygous polled Holstein sire used in artificial insemination with a polled mother and polled grandmother that was heterozygous for the SNP *AC000158: g.1390292G>A*. This animal carried neither the previously published 202-bp-indel associated with polledness in cattle [15] nor the polled-associated alleles from three tested markers (P5ID, PC1768587A and P80kbID) belonging to the previously published polled-associated haplotype of seven polymorphisms [15]. Thus, this sire would not have been identified as genetically polled without the SNP *AC000158: g.1390292G>A*. Furthermore, there was one homozygous polled pure-bred Holstein that was a compound heterozygote for the polled and horned alleles at both the seven polymorphism haplotype and 202 bp indel loci predictive of polled [15]. However, this animal was homozygous A/A for the SNP *AC000158: g.1390292G>A*, and was therefore identified reliably as homozygous polled by only genotyping this SNP.

The SNP *AC000158: g.1385927G>A* in intron 4 was also significantly associated with the horned and polled genotypes in the initially analysed animals (χ^²^=13, -log_10_P=2.82). Association of this SNP could not be confirmed analysing an extended number of Holsteins. All three genotypes were found in the analysed homozygous polled animals. This SNP was thus not genotyped in all horned animals.

The indels *AC000158: g.1394768_771delCTGC* and *AC000158: g.1400851_852delCT* showed significant association (Table 1 Figure 1) but since they did not perfectly co-segregate with the horned and polled phenotype as did the SNP *AC000158: g.1390292G>A*, they were genotyped in all polled but not all of the horned animals.

SNPs within the coding sequence of exons 3 and 7 (*AC000158: g.1376880T>A, AC000158: g.1376884T>C, AC000158: g.1376932A>G, AC000158: g.1390505T>C* and *AC000158: g.1390528T>C*) and the SNP *AC000158: g.1376520T>C* within the 3’ UTR were not associated with polledness in Holsteins (Table 1).

**Figure 2:**
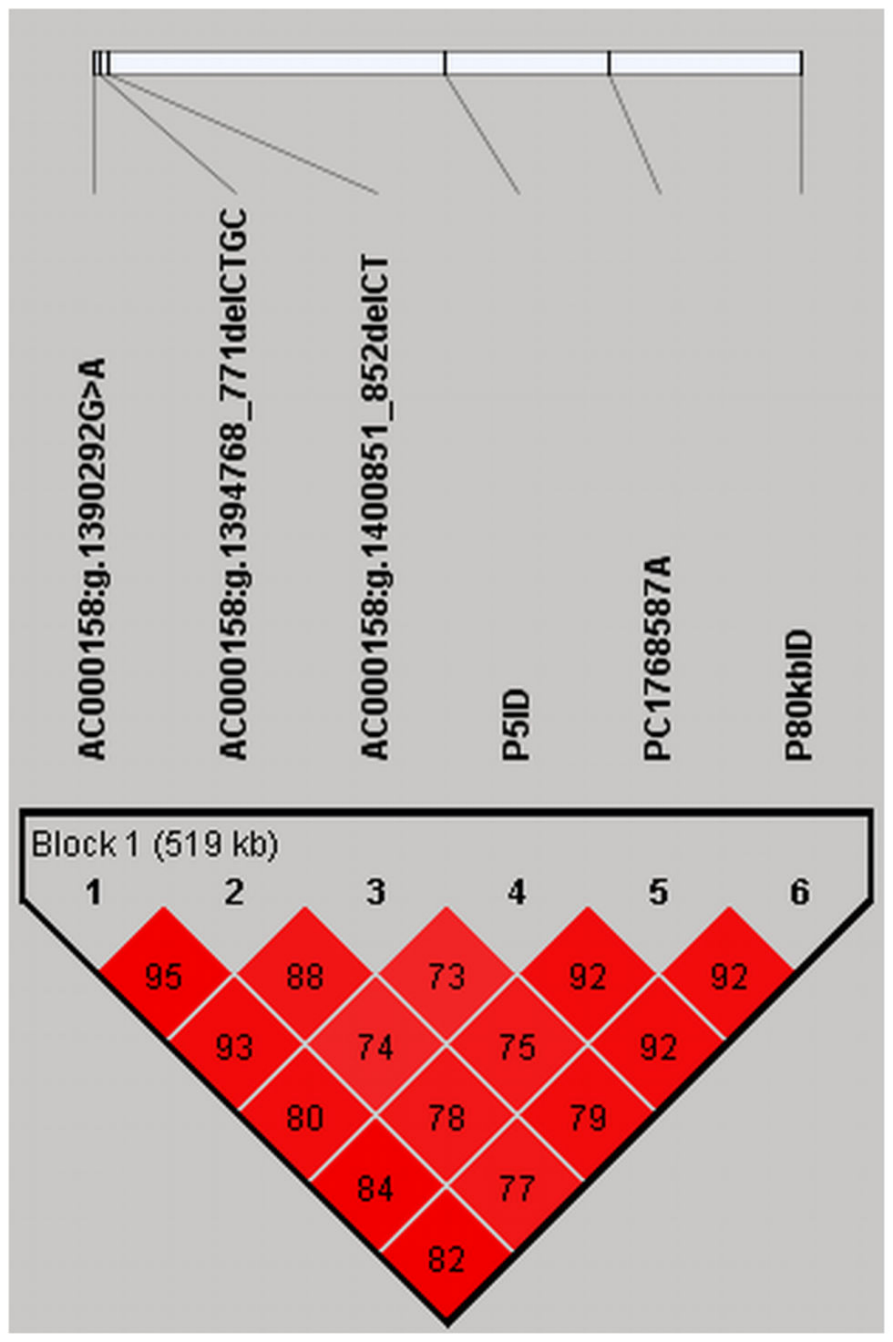
Linkage disequilibrium among markers within the 1 Mb BTA1 region associated with polledness in Holsteins. *AC000158: g.1390292G>A*, *AC000158: g.1394768_771delCTGC* and *AC000158: g.1400851_852delCT* are newly discovered polymorphisms located in the *IFNGR2* gene. P5ID, PC1768587A and P80kbID represent three markers of the polled-haplotype previously published for Holstein [15]. LD coefficients (*r* ^*2*^) between pairs of polymorphisms are indicated as black letters in red fields. All pictured polymorphisms share one common haplotype block of 519 kb length.

The *r* ^2^-values indicating correlation among mutated alleles showed that the three polymorphisms *AC000158: g.1390292G>A*, *AC000158: g.1394768_771delCTGC* and *AC000158: g.1400851_852delCT* are in a strong linkage disequilibrium (LD) (Figure 2). Although these three mutations are located over 200 kb proximal on BTA1 to the three markers P5ID, PC1768587A and P80kbID [15], all six polymorphisms form a haplotype block of 519 kb length (Figure 2).

## Discussion

Of the examined genes *MRPS6*, *SLC5A3*, *ATP5O*, *ITSN1*, *CRYZL1*, *DONSON*, *SON*, *GART*, *LOC784171*, *TMEM50B*, *IFNGR2*, *IFNAR1*, *IL10RB*, *IFNAR2*, *HIST1H4C* and *OLIG1* and the pseudogenes *40S ribosomal protein S13-like* and *OLIG2-like* within the 1-Mb-polled-region on BTA1, the highest association with polledness in Holsteins was found within the *interferon gamma* receptor *2* gene (*IFNGR2*). Within this gene, we detected the SNP *AC000158: g.1390292G>A* that perfectly cosegregates with polledness in Holsteins.

*IFNGR2* encodes the non-ligand-binding beta chain of the gamma interferon receptor which is involved in immunological pathways. There are currently no known functions of the *IFNGR2* gene that could play a role in the growth of horns.

The SNP *AC000158: g.1390292G>A* is unlikely to have a causal function for polledness in Holsteins, as it is located in a noncoding region of the *IFNGR2* gene (intron 3) and effects on gene regulating elements were not found. The other associated polymorphisms (*AC000158: g.1394768_771delCTGC* and *AC000158: g.1400851_852delCT*) are also located within introns. The indel *AC000158: g.1400851_852delCT* in intron 1 was associated with polledness and disrupts a transcription factor binding site (Table 1) but the affected factor ICSBP has no distinguishable role in the development of horns and the distribution of alleles does not discriminate the polled genotype in the analysed animals. *AC000158: g.1394768_771delCTGC* and *AC000158: g.1400851_852delCT*, therefore, do not seem to have a causal function, either.

SNPs within exon 3 of *IFNGR2* (*AC000158: g.1390505T>C* and *AC000158: g.1390528T>C*) led to amino acid changes but were predicted to be benign. Only a SNP *AC000158: g.1376880T>A* causing an amino acid change from threonine to serine on cDNA position 338 was evaluated as probably damaging to protein function using PolyPhen-2 but failed the significance for association with polledness.

The SNP AC000158: g.1376520T>C within the 3’ UTR disrupts a binding site for the factor AP-1 (Table 1), which is involved in cell transformation, inflammation, innate immune response and cell death. However, this SNP was not associated with polledness.

*AC000158: g.1376880T>A* causing an amino acid change within the encoded protein shows no significant associations with polledness in cattle. Thus, the coding sequence of *IFNGR2* is unlikely to exert an influence on the development of horns. MicroRNAs located within the *IFNGR2-gene* were not found in the human and mouse database miRBase (http://www.mirbase.org/). High conservation of microRNA-regions in various species is often observed [16]. Multiple sequence alignment including the species cow, mouse, human and sheep was performed using the program ClustalW2 (http://www.ebi.ac.uk/Tools/msa/clustalw2/). For intron 3 of *IFNGR2* harbouring the highly associated SNP *AC000158: g.1390292G>A* identity-values from 59% between the species human and cow to 90% between the species cow and sheep were found.

However, there could be mutations within currently unknown regulatory regions nearby or within the *IFNGR2* gene or mutations within the 1-Mb-polled-region that interact with the *IFNGR2* gene that may cause polledness in Holsteins. Thus, the *IFNGR2* gene cannot be excluded as a potential candidate gene for polledness in cattle.

Nevertheless, the SNP *AC000158: g.1390292G>A* showed perfect co-segregation with polledness in Holsteins. Nearly 90% of the examined Holsteins have a multinational background with ancestors from at least one of 11 different countries. Despite of that their polled genotype could be determined reliably by analysing the SNP *AC000158: g.1390292G>A*. The genotype of one heterozygous polled Holstein sire could be confirmed using this SNP, while this animal neither carried the previously published haplotype block nor the 202-bp-indel associated with polledness in cattle [15]. One homozygous polled Holstein that was a compound heterozygous for the haplotype block of seven polymorphisms and the 202-bp-indel [15] was revealed to be homozygous polled by the SNP *AC000158: g.1390292G>A*. Thus, the SNP *AC000158: g.1390292G>A* can be used as single polymorphism for testing of the polled genotype of pure-bred Holsteins, independent of their origin.

The *IFNGR2* gene is located within the 1-Mb-polled-region but more than 200 kb proximal to the published homozygosity region [13]. The high association of the SNP *AC000158: g.1390292G>A* within intron 3 of *IFNGR2* indicates that the causal polled mutation is not necessarily located within this homozygous region of 381 kb. Performing homozygosity mapping, small associated regions can be missed due to the distribution of SNPs examined using the BovineSNP50 BeadChip (Illumina) if these small regions are located in-between the genotyped SNPs. Furthermore, homozygosity mapping across breeds would not be successful if the polled mutation is non-allelic among the different polled breeds. The candidate region for polled therefore still contains the entire non-recombinant region of approximately 1 Mb between the microsatellites *RP42-218J17_MS1* (661 kb) and *BM6438* (2,025 kb) [9].

## Conclusions

Herein, we demonstrate a single SNP as a useful and reliable marker for polledness in Holsteins, independent of their origin. We furthermore propose that the whole well-established candidate interval that showed no recombination in polled animals from 661 kb to 2,025 kb on BTA1 should be scanned to find the causal polled mutation.

## Materials and Methods

### Ethics statement

All animal work has been conducted according to the national and international guidelines for animal welfare. The Lower Saxony state veterinary office at the Niedersächsisches Landesamt für Verbraucherschutz und Lebensmittelsicherheit, Oldenburg, Germany, was the responsible Institutional Animal Care and Use Committee (IACUC) for this specific study. The EDTA-blood and semen sampling for the present study had been approved by the IACUC of Lower Saxony, the state veterinary office at the Niedersächsisches Landesamt für Verbraucherschutz und Lebensmittelsicherheit, Oldenburg, Germany (registration number 02A/101).

### Animals, phenotypic data and DNA preparation

EDTA preserved blood samples of three male and one female homozygous polled, five male and five female heterozygous polled and two male and 19 female horned, not closely related pure-bred Holsteins were used for the detection of new polymorphisms. The genotypes of the homozygous polled animals were proved by progeny-testing. The heterozygous polled animals were each offspring of a polled sire and a horned dam.

For genotyping of polymorphisms with highest association with polledness we used further EDTA preserved blood samples and semen samples of 11 homozygous polled, 61 heterozygous and 336 horned unrelated Holsteins of highly diverse origin (Figures S2-S3).

In addition, we used EDTA preserved blood samples of 21 homozygous polled, 43 heterozygous polled and 28 horned German Fleckvieh and five homozygous polled, nine heterozygous polled and eight horned Charolais. Bovine genomic DNA was isolated from EDTA preserved blood using the NucleoSpin 96 Blood Quick Pure Kit (Macherey Nagel, Düren, Germany) or from EDTA preserved blood samples or semen samples following a standard protocol with ethanol-precipitation.

### Mutation analyses

Primers for PCR were designed using primer3 (http://frodo.wi.mit.edu/) covering intergenic regions and the genes *MRPS6*, *SLC5A3*, *ATP5O*, *ITSN1*, *CRYZL1*, *DONSON*, *SON*, *GART*, *LOC784171*, *TMEM50B*, *IFNGR2*, *IFNAR1*, *IL10RB*, *IFNAR2*, *HIST1H4C* and *OLIG1* and the pseudogenes *LOC782947* and *OLIG2-like* (Table S1). Repetitive regions in sequence-data were masked using RepeatMasker (http://www.repeatmasker.org/) before developing the primers. In addition, we applied Primer-BLAST (http://www.ncbi.nlm.nih.gov/tools/primer-blast) to ensure that the designed primer sequences were unique within the bovine genome. PCR-products were directly sequenced. PCR reactions were performed in a total volume of 32 μl using 2 μl (~ 20 ng/µl) genomic DNA, 3.2 μl 10x PCR buffer, 0.62 μl DMSO, 0.62 μl dNTPs (10 mM each), 0.18 μl (100 pmol/μl) of each primer and 0.18 μl Taq Polymerase (MP Biomedicals, Eschwege, Germany). The PCR conditions were as follows: 5 min initial denaturation at 95°C, followed by 36 cycles at 95°C for 30 sec, optimal annealing temperature (T_a_, differed between 57°C and 60°C, according to the primer) for 1 min and extension at 72°C for 45 sec. The PCR was completed with a final cooling at 4°C for 10 min.

The amplicons were purified using the MinElute 96 UF Plate (Qiagen, Hilden, Germany) or ExoSAP-IT (Affymetrix, München, Germany) and directly sequenced with the DYEnamic ET Terminator Cycle Sequencing kit (GE Healthcare, Freiburg, Germany) on a MegaBACE 1000 capillary sequencer (GE Healthcare) or an ABI Prism 3500 capillary sequencer (Applied Biosystems, Darmstadt, Germany). Determined sequence data was analyzed using the Sequencher 4.8 program (GeneCodes, Ann Arbor, MI) and screened for polymorphisms.

Additionally, the genomes of one homozygous polled and three horned Holsteins and two DNA-pools, respectively, with six horned Holsteins each were sequenced using paired-end sequencing on the Illumina HiSeq 2000 (LGC Genomics, Berlin, Germany). On the average, an eight-fold coverage per sample was accomplished.

### Genotyping

Genotyping of SNPs was performed by sequencing as described above. SNPs *AC000158: g.1390292G>A* and *AC000158: g.1385927G>A* were genotyped by PCR-RFLP (restriction fragment length polymorphism) or mismatch PCR-RFLP [17], respectively. The restriction enzymes were selected from the NEBcutter V 2.0 (http://tools.neb.com/NEBcutter2/). For SNP *AC000158: g.1390292G>A* the restriction enzyme Hha1 was used. For genotyping of SNP *AC000158: g.1385927G>A* we used the restriction enzyme DdeI. Digested products were size-fractionated by gel electrophoresis on 2% to 4% agarose gels (peqGOLD Universal Agarose, Peqlab Biotechnologie, Erlangen, Germany).

Indel mutations were amplified by using DY-682-tagged primers (Eurofins MWG Operon, Ebersberg, Germany). PCR reactions were performed in a total volume of 12 μl using 2 μl (~ 20 ng/µl) genomic DNA, 1.2 μl 10x PCR buffer, 0.4 μl DMSO, 0.2 μl dNTPs (10mM each), 0.5 μl (10 pmol/μl) of each primer and 0.2 μl Taq Polymerase (MP Biomedicals). The PCR conditions were as follows: 5 min initial denaturation at 94°C, followed by 36 cycles at 94°C for 30 sec, 58°C to 60°C (optimal annealing temperature according to the primer) for 1 min and extension at 72°C for 30 sec. The PCR was completed with a final cooling at 4°C for 10 min. Afterwards the PCR products were analysed by gel electrophoresis on the automated sequencer LI-COR 4300 (LI-COR, Bad Homburg, Germany) using 4% to 6% polyacrylamide denaturing gels (Rotiphorese Gel40, Carl Roth, Karlsruhe, Germany). Differences in allele sizes caused by deleted or inserted bases were defined by visual examination.

### Statistical analyses

Association analysis with polledness in cattle was carried out for the detected polymorphisms. The markers have been tested for their polymorphism information content, Hardy-Weinberg-Equilibrium (HWE) and association with the polled phenotype and the polled genotype using the procedures ALLELE and CASECONTROL of SAS/Genetics, Version 9.3 (Statistical Analysis System, Cary, NC, 2012). Statistical calculation of pairwise linkage disequilibrium (LD) was performed and pictured using HAPLOVIEW 4.2 (http://www.broad.mit.edu/mpg/haploview/). For detection of SNPs with strong LD among the alleles we used the tagger algorithm *r* ^2^≥0.8[18]. . 

### In silico screening for binding sites of gene-regulating elements

Polymorphisms associated with polledness and mutations located within the UTR-regions were screened for effects on binding sites for gene regulating elements using AliBaba 2.1 (http://www.gene-regulation.com/pub/programs/alibaba2/index.html).

SNPs localised in coding sequence were examined for causing amino acid changes. Discovered changes in amino acid order were analysed using PolyPhen-2 (http://genetics.bwh.harvard.edu/pph2) with regard to their influence on protein function.

The expression of genes can also be regulated post-transcriptionally by microRNAs (small non-coding RNAs) [19]. In vertebrates 40-70% of microRNAs are expressed from introns [20]. The database miRBase (http://www.mirbase.org/) was therefore scanned for microRNAs contained inside the *IFNGR2-gene* in human and mouse.

## Supporting Information

Figure S1 Gene structure of *IFNGR2* with PCR-products and all polymorphisms. (**a**) Rectangles indicate exons. Coding sequence is filled with black, the untranslated region is filled with white. The size of exons and introns is specified in number of base pairs (bp). The black lines below illustrate the sections analysed in Holsteins. Each PCR-product is pictured separately with start and ending position. Position of start and stop codon is stated in bp. All polymorphisms in Holsteins are given with name and position. Each position is given with accordance to Bos taurus assembly UMD3.1. (**b**) Coverage-data for the *IFNGR2* gene sequenced in the course of whole genome sequencing using the Illumina HiSeq 2000 (LGC).
(DOC)

Figure S2 Pedigrees of two heterozygous polled Holstein sires. For each ancestor the birth-country and the polled genotype are given, if known. (**a**) Heterozygous polled Holstein sire born in Germany with ancestors from United States on the mother’s side and a British father with ancestors from Italy and Canada. (**b**) Heterozygous polled Holstein sire born in Belgium but with German herdbook number. The animal has ancestors from the Netherlands and Austria on the mother’s side and a Canadian father with US ancestors.
(DOC)

Figure S3 Countries of birth of 77 of the 81 polled Holstein sires. The countries are each drawn in a different colour. The distribution is given in per cent.
(DOC)

Table S1 PCR-primers used for mutation analyses. For each primer localisation, name, the sequence, annealing temperature and product sizes in base pairs (bp) are given.
(DOC)

Table S2 Polymorphisms located in the bovine *interferon* *gamma* receptor *2* (*IFNGR2*) on BTA1 in Holsteins. (DOC)

## References

[1] Lange H (1989) Untersuchungen über Hornlosigkeit und Kopfform beim deutschen Fleckvieh. Inaugural-Dissertation. Munich: Veterinary Faculty of the Ludwig-Maximilians-University.

[2] White WT, Ibsen H (1936) Horn inheritance in Galloway-Holstein cattle crosses. J Genet 32: 33-49. doi:10.1007/BF02982500.

[3] Long CR, Gregory KE (1978) Inheritance of the horned, scurred, and polled condition in cattle. J Hered 69: 395-400.

[4] Brem G, Karnbaum B, Rosenberg E (1982) Zur Vererbung der Hornlosigkeit beim Fleckvieh. Bayer Landwirtsch Jahrb 59: 688-695.

[5] Georges M, Drinkwater R, Lefort A, Libert F, King T et al. (1993) Microsatellite mapping of a gene affecting horn development in Bos taurus. Nat Genet 4: 206-210. doi:10.1038/ng0693-206. PubMed: 8348158. 8348158 10.1038/ng0693-206

[6] Schmutz SM, Marquess FL, Berryere TG, Moker JS (1995) DNA marker-assisted selection of the polled condition in Charolais cattle. Mamm Genome 6: 710-713. doi:10.1007/BF00354293. PubMed: 8563169. 8563169 10.1007/BF00354293

[7] Harlizius B, Tammen I, Eichler K, Eggen A, Hetzel DJ (1997) New markers on bovine chromosome 1 are closely linked to the polled gene in Simmental and Pinzgauer cattle. Mamm Genome 8: 255-257. doi:10.1007/s003359900404. PubMed: 9096105. 9096105 10.1007/s003359900404

[8] Drögemüller C, Bader A, Wöhlke A, Kuiper H, Leeb T et al. (2002) A high-resolution comparative RH map of the proximal part of bovine chromosome 1. Anim Genet 33: 271-279. doi:10.1046/j.1365-2052.2002.00866.x. PubMed: 12139506. 12139506 10.1046/j.1365-2052.2002.00866.x

[9] Drögemüller C, Wöhlke A, Mömke S, Distl O (2005) Fine mapping of the polled locus to a 1-Mb region on bovine chromosome 1q12. Mamm Genome 16: 613-620. doi:10.1007/s00335-005-0016-0. PubMed: 16180143. 16180143 10.1007/s00335-005-0016-0

[10] Threadgill DS, Kraus JP, Krawetz SA, Womack JE (1991) Evidence for the evolutionary origin of human chromosome 21 from comparative gene mapping in the cow and mouse. Proc Natl Acad Sci U S A 88: 154-158. doi:10.1073/pnas.88.1.154. PubMed: 1986361. 1986361 10.1073/pnas.88.1.154 PMC50768

[11] Chowdhary BP, Frönicke L, Gustavsson I, Scherthan H (1996) Comparative analysis of the cattle and human genomes: detection of ZOO-FISH and gene mapping-based chromosomal homologies. Mamm Genome 7: 297-302. doi:10.1007/s003359900086. PubMed: 8661702. 8661702 10.1007/s003359900086

[12] Stella A, Ajmone-Marsan P, Lazzari B, Boettcher P (2010) Identification of selection signatures in cattle breeds selected for dairy production. Genetics 185: 1451-1461. doi:10.1534/genetics.110.116111. PubMed: 20479146. 20479146 10.1534/genetics.110.116111 PMC2927769

[13] Seichter D, Russ I, Rothammer S, Eder J, Förster M et al. (2012) SNP-based association mapping of the polled gene in divergent cattle breeds. Anim Genet 43: 595-598. doi:10.1111/j.1365-2052.2011.02302.x. PubMed: 22497248. 22497248 10.1111/j.1365-2052.2011.02302.x

[14] Mariasegaram M, Reverter A, Barris W, Lehnert SA, Dalrymple B et al. (2010) Transcription profiling provides insights into gene pathways involved in horn and scurs development in cattle. BMC Genomics 11: 370. doi:10.1186/1471-2164-11-370. PubMed: 20537189. 20537189 10.1186/1471-2164-11-370 PMC3017764

[15] Medugorac I, Seichter D, Graf A, Russ I, Blum H et al. (2012) Bovine polledness--an autosomal dominant trait with allelic heterogeneity. PLOS ONE 7: e39477. doi:10.1371/journal.pone.0039477. PubMed: 22737241. 22737241 10.1371/journal.pone.0039477 PMC3380827

[16] Kim KS, Kim JS, Lee MR, Jeong HS, Kim J (2009) A study of microRNAs in silico and in vivo: emerging regulators of embryonic stem cells. FEBS J 276: 2140-2149. doi:10.1111/j.1742-4658.2009.06932.x. PubMed: 19250314. 19250314 10.1111/j.1742-4658.2009.06932.x

[17] Quadros L, Ghosh K, Shetty S (2008) Establishment of a new mismatch PCR-RFLP technique for detection of G10430A common mutation present in moderate to mild haemophilia B patients belonging to Gujarati community from the western part of India. Haemophilia 14: 628-629. doi:10.1111/j.1365-2516.2008.01704.x. PubMed: 18393981. 18393981 10.1111/j.1365-2516.2008.01704.x

[18] de Bakker PI, Yelensky R, Pe’er I, Gabriel SB, Daly MJ et al. (2005) Efficiency and power in genetic association studies. Nat Genet 37: 1217-1223. doi:10.1038/ng1669. PubMed: 16244653. 16244653 10.1038/ng1669

[19] Ma C, Liu Y, He L (2009) MicroRNAs - powerful repression comes from small RNAs. Sci China C 4: 323-330. PubMed: 19381458. 10.1007/s11427-009-0056-x PMC3681298 19381458

[20] Griffiths-Jones S, Saini HK, van Dongen S, Enright AJ (2008) miRBase: tools for microRNA genomics. Nucleic Acids Res 36: D154-D158. doi:10.1093/nar/gkn221. PubMed: 17991681. 17991681 10.1093/nar/gkm952 PMC2238936

