# Supplementary material for: A Single Nucleotide Polymorphism within the Interferon Gamma Receptor 2 Gene Perfectly Coincides with Polledness in Holstein Cattle
Source: PLoS One. 2013 Jun 21;8(6):e67992. doi: 10.1371/journal.pone.0067992 (PMC3689702; doi:10.1371/journal.pone.0067992)
Supplement: Figure S1 — (a) Rectangles indicate exons. Coding sequence is filled with black, the untranslated region is filled with white. The size of exons and introns is specified in number of base pairs (bp). The black lines below illustrate the sections analysed in Holsteins. Each PCR-product is pictured separately with start and ending position. Position of start and stop codon is stated in bp. All polymorphisms in Holsteins are given with name and position. Each position is given with accordance to Bos taurus assembly UMD3.1. (b) Coverage-data for the IFNGR2 gene sequenced in the course of whole genome sequencing using the Illumina HiSeq 2000 (LGC). (DOC) [file pone.0067992.s001.doc]

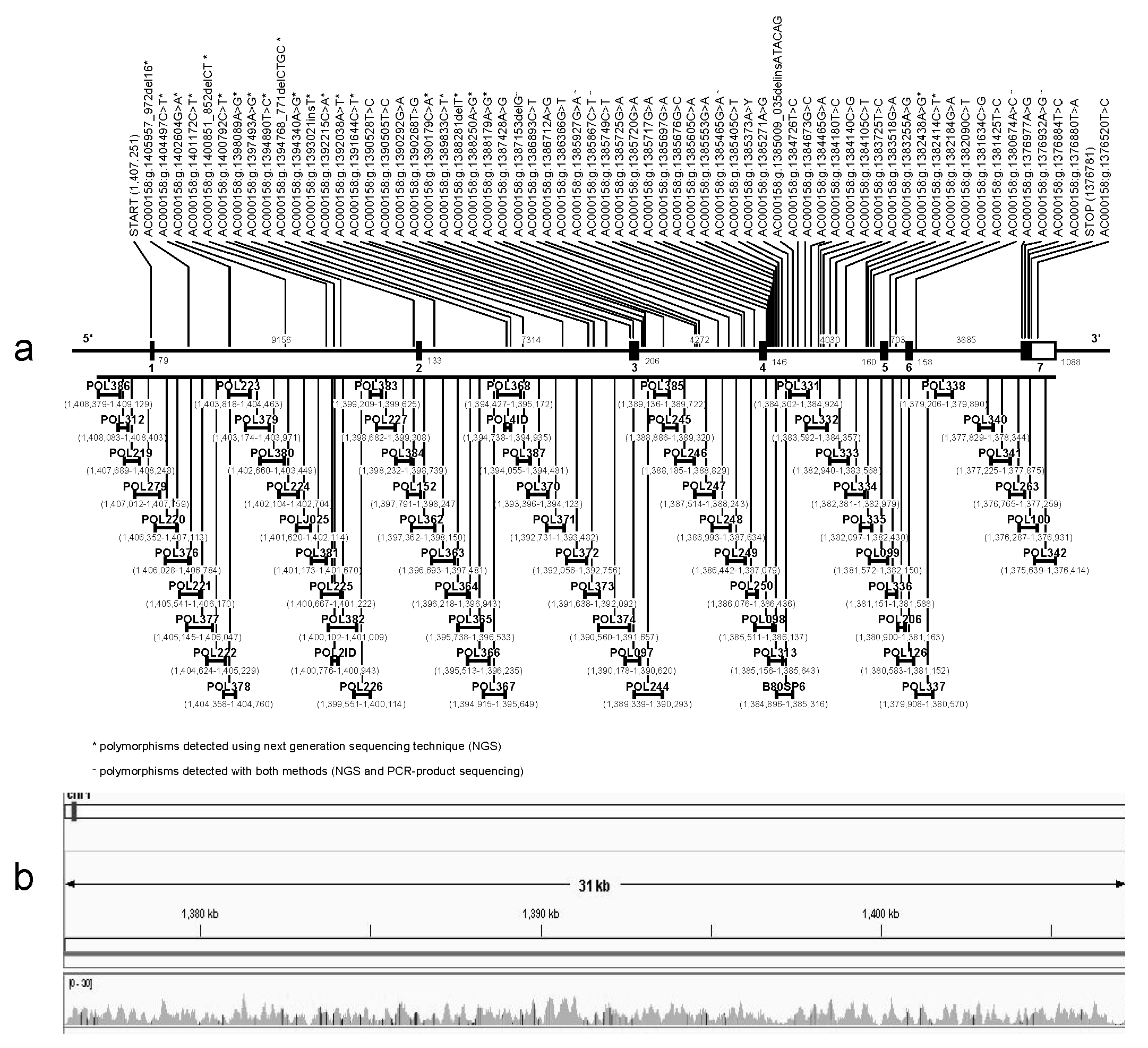


**Figure S1.** **Gene structure of *IFNGR2* with PCR-products and all polymorphisms.** (**a**) Rectangles indicate exons. Coding sequence is filled with black, the untranslated region is filled with white. The size of exons and introns is specified in number of base pairs (bp). The black lines below illustrate the sections analysed in Holsteins. Each PCR-product is pictured separately with start and ending position. Position of start and stop codon is stated in bp. All polymorphisms in Holsteins are given with name and position. Each position is given with accordance to Bos taurus assembly UMD3.1. (**b**) Coverage-data for the *IFNGR2* gene sequenced in the course of whole genome sequencing using the Illumina High Seq 2000 (LGC).
