# Supplementary material for: A Single Nucleotide Polymorphism within the Interferon Gamma Receptor 2 Gene Perfectly Coincides with Polledness in Holstein Cattle
Source: PLoS One. 2013 Jun 21;8(6):e67992. doi: 10.1371/journal.pone.0067992 (PMC3689702; doi:10.1371/journal.pone.0067992)
Supplement: Figure S2 — For each ancestor the birth-country and the polled genotype are given, if known. (a) Heterozygous polled Holstein sire born in Germany with ancestors from United States on the mother’s side and a British father with ancestors from Italy and Canada. (b) Heterozygous polled Holstein sire born in Belgium but with German herdbook number. The animal has ancestors from the Netherlands and Austria on the mother’s side and a Canadian father with US ancestors. (DOC) [file pone.0067992.s002.doc]

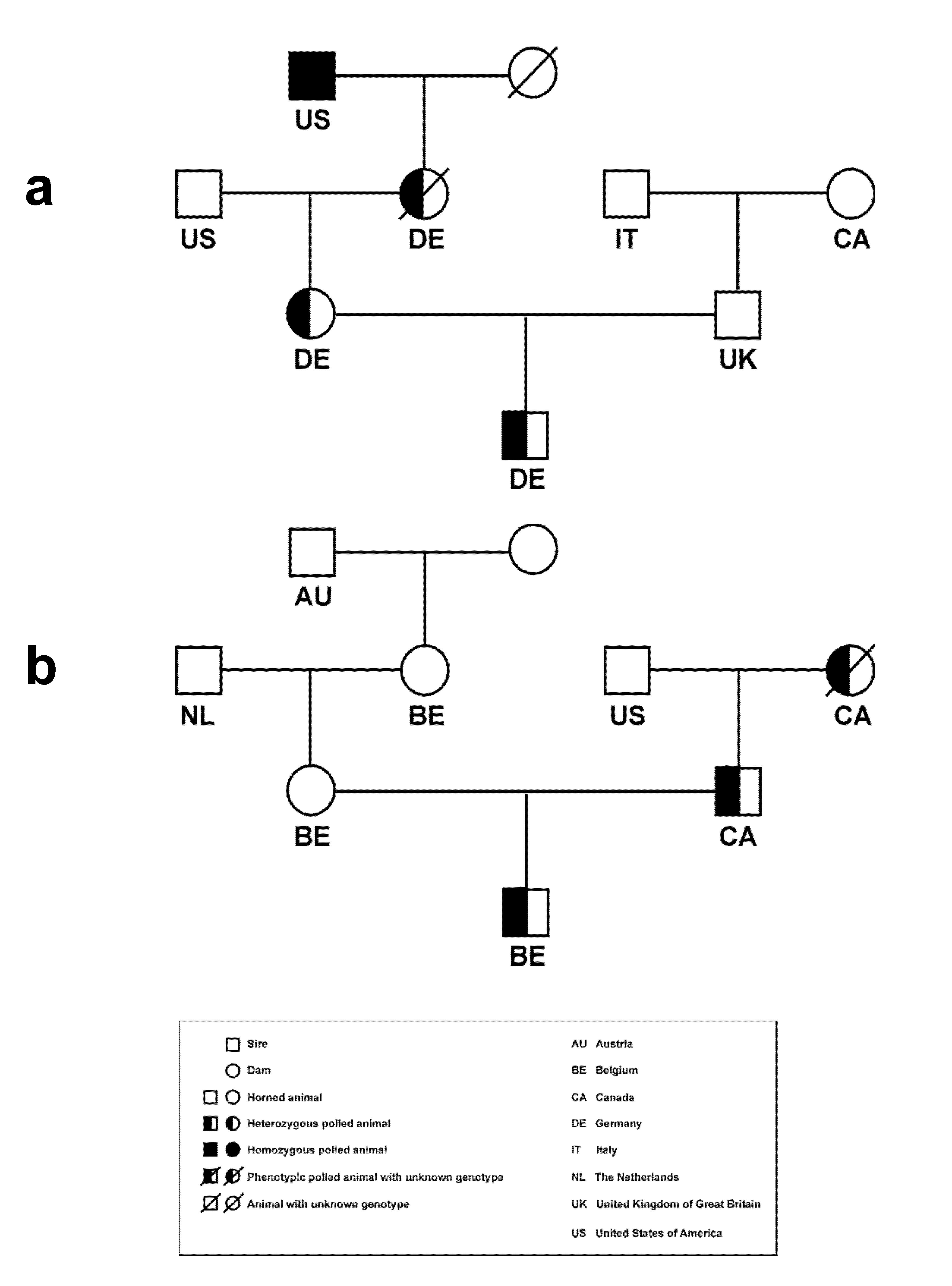


**Figure S2. Pedigrees of two heterozygous polled Holstein sires.** For each ancestor the birth-country and the polled genotype are given, if known. (**a**) Heterozygous polled Holstein sire born in Germany with ancestors from United States on the mother’s side and a British father with ancestors from Italy and Canada. (**b**) Heterozygous polled Holstein sire born in Belgium but with German Stud-book number. The animal has ancestors from the Netherlands and Austria on the mother’s side and a Canadian father with US ancestors.
