# Supplementary material for: A Single Nucleotide Polymorphism within the Interferon Gamma Receptor 2 Gene Perfectly Coincides with Polledness in Holstein Cattle
Source: PLoS One. 2013 Jun 21;8(6):e67992. doi: 10.1371/journal.pone.0067992 (PMC3689702; doi:10.1371/journal.pone.0067992)
Supplement: Figure S3 — The countries are each drawn in a different colour. The distribution is given in per cent. (DOC) [file pone.0067992.s003.doc]

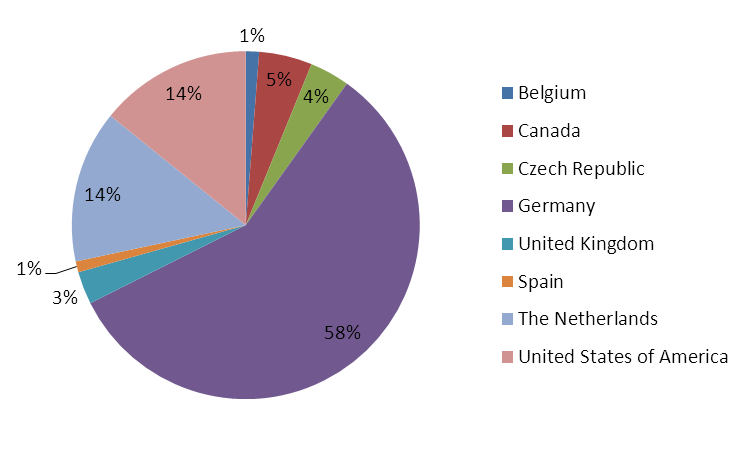


**Figure S3. Countries of birth of 77 of the 81 polled Holstein sires.** The countries are each drawn in a different colour. The distribution is given in per cent.
