# Supplementary material for: A Single Nucleotide Polymorphism within the Interferon Gamma Receptor 2 Gene Perfectly Coincides with Polledness in Holstein Cattle
Source: PLoS One. 2013 Jun 21;8(6):e67992. doi: 10.1371/journal.pone.0067992 (PMC3689702; doi:10.1371/journal.pone.0067992)
Supplement: Table S1 — For each primer localisation, name, the sequence, annealing temperature and product sizes in base pairs (bp) are given. (DOC) [file pone.0067992.s004.doc]

**Table S1 PCR-primers used for mutation analyses.** For each primer localisation, name, sequence, annealing temperature and product sizes in base pairs (bp) are given.

| Gene ID | Target within the gene  (assembly UMD3.1) | Primer | Sequence of primers  (5’ – 3’) | Annealing temperature (°C) | Product size (bp) |
| --- | --- | --- | --- | --- | --- |
| *MRPS6* | Exon1 | POL266_F | GTAGCCAGCGCAGGGTCT | 60 | 507 |
|  |  | POL266_R | CTGCATGGCTTTCAAGATCA |  |  |
|  | Intron1 | POL278_F | CGTCAGTTGGAGGGTCTTTG | 60 | 652 |
|  |  | POL278_R | CCTCACCTGCCTCAAATCAT |  |  |
|  |  | POL267_F | TTGCAGGTATTGTTGTCATGC | 60 | 626 |
|  |  | POL267_R | CCAATGGAAGACTGCTGGAG |  |  |
|  |  | POL273_F | AAAGGCATGGAGAAGGGTTT | 60 | 644 |
|  |  | POL273_R | GCAAGGAGAGGAGGGAAAAA |  |  |
|  |  | POL274_F | GGCCTGTTGCTTGATGAAAT | 60 | 615 |
|  |  | POL274_R | TCTGACGTCTGCTCAGGCTA |  |  |
|  |  | POL272_F | TATGGAATGTTGGGGGAAAG | 60 | 559 |
|  |  | POL272_R | ATGCAGAGGCTGCGTACATA |  |  |
|  |  | POL317_F | GTTTGCCAGGTTCGTTTTGT | 59 | 536 |
|  |  | POL317_R | TCTAATCAAGTGGGGCAACC |  |  |
|  |  | POL318_F | CCTTAGTGGATCCATTGGAAGA | 59 | 686 |
|  |  | POL318_R | TTTTCCCCTAAAGAATACAAGGTT |  |  |
|  |  | POL319_F | ATTCCCCTTCATGAGCACAG | 59 | 598 |
|  |  | POL319_R | CCAGACTTGGACACCTGGTT |  |  |
|  |  | POL320_F | TAATAAGCTGGGGGCCAAAT | 59 | 850 |
|  |  | POL320_R | TCTTAGCTTGAGCCAATAAGAAAA |  |  |
|  |  | POL321_F | GGGTCCTAAGTGATGACATTGA | 59 | 640 |
|  |  | POL321_R | CAACCTTCCTCCCCAATTCT |  |  |
|  |  | POL322_F | CCTGGGTGTAGCAGCCATAG | 59 | 679 |
|  |  | POL322_R | TCTTACATAATCCAGCTTCACACC |  |  |
|  |  | POL323_F | GCCTTGTTGCAGCAATTAAA | 59 | 729 |
|  |  | POL323_R | TCCAGGTAGCCAAATCGAAC |  |  |
|  |  | POL324_F | TTTTGGAGGGAGGACCATAA | 59 | 608 |
|  |  | POL324_R | ACCCTACAGAATGGCCTGTG |  |  |
|  |  | POL325_F | TCAGTGTCGCCTATACCTCTG | 59 | 669 |
|  |  | POL325_R | ACCCATGGCAGGCTTCTTAT |  |  |
|  |  | POL326_F | GTAACTCCGCGTGTGGAAAG | 59 | 677 |
|  |  | POL326_R | CACAACAGGCAAAAGAATCC |  |  |
|  |  | POL327_F | GCAGAATTCGATCCAGGTGT | 59 | 627 |
|  |  | POL327_R | CTCCTTTGCCTTATCCCACA |  |  |
|  |  | POL328_F | GCTTTGGTGGAGTTGAGAGG | 59 | 626 |
|  |  | POL328_R | AGCAGCAGCACTGAGAAACA |  |  |
|  |  | POL329_F | TGCTTCAGTTGATGGATTGC | 59 | 633 |
|  |  | POL329_R | GATGGACTTGGTAAGTCAAACCTT |  |  |
|  |  | POL330_F | CAGTGTACCCTTCATTGTCTGC | 59 | 624 |
|  |  | POL330_R | AAAGGAACTGAGGCGCAAC |  |  |
|  | Exon2 | POL268_F | ACCCAAAGATCCCTTCCAAT | 60 | 514 |
|  |  | POL268_R | GGATTCCCACAATCAGAAGC |  |  |
|  | Intron2 | POL276_F | TTTTCCTGGCCTGTTTTGTC | 60 | 629 |
|  |  | POL276_R | CAGCGTCATCAAGCCTTTCT |  |  |
|  | Exon3 | POL269_F | ATCGCTGTATTTCCGAATGC | 60 | 516 |
|  |  | POL269_R | GGGGTTAGTGTTGCTGTTGG |  |  |
|  |  | POL270_F | GCTGTTGGATTTTCCAGAGG | 60 | 502 |
|  |  | POL270_R | GCTAGTGGCAGAGGAACGAC |  |  |
|  | 3’flanking region | POL277_F | TAAAGACCCTTGGCCTCTTG | 60 | 615 |
|  |  | POL277_R | CCAACTTCTCCTCTGCCAAC |  |  |
|  |  | B332_T7_F | GGTTGAGAGAAGAAGGGCTTG | 59 | 143 |
|  |  | B332_T7_R | CAGAGAAAGCAAAGCTGAGAAG |  |  |
| *SLC5A3* | 5’flanking region | POL264_F | CGAATACAAGCGTCCAATGA | 60 | 595 |
|  |  | POL264_R | GCAGTTGTTGTTGCCAGAGA |  |  |
|  |  | POLJ047_F | CTGGCAATCCTGGGTTAGAA | 60 | 638 |
|  |  | POLJ047_R | AAGCACACAGCAGAGGCATA |  |  |
|  | Exon1 | POL169_F | TGCGAAATAGACCTGAATCC | 58 | 476 |
|  |  | POL169_R | CGTATGGTTTCCAGACTTGG |  |  |
|  |  | POL168_F | ACACCAGTACCAGACTGAAGC | 57 | 560 |
|  |  | POL168_R | AACTTCTGGGATGGGTTTTC |  |  |
|  |  | POL167_F | ATCTGGCCTCCTTGCATC | 58 | 594 |
|  |  | POL167_R | TAATGTCCACCCCAAGAAAG |  |  |
|  |  | POL166_F | CCTCAGACTTCCCGTTGG | 59 | 598 |
|  |  | POL166_R | GTGTACAAACTCATCCGCAAG |  |  |
|  |  | POL165_F | AATGCCTGAGAGATGCACAG | 60 | 599 |
|  |  | POL165_R | AGAGCTGCTCCCCGAAAG |  |  |
|  | 3’untranslated region | POL265_F | CCTCTTCCTTTCTTCCAGCA | 60 | 562 |
|  |  | POL265_R | TGGTCTTTGGAAAACAACAACA |  |  |
|  |  | POLJ020_F | TTTTCCCAGAGATGGATGAAA | 60 | 385 |
|  |  | POLJ020_R | ACAAAACGAACCTGGCAAAC |  |  |
|  |  | POL272_F | TATGGAATGTTGGGGGAAAG | 60 | 559 |
|  |  | POL272_R | ATGCAGAGGCTGCGTACATA |  |  |
|  |  | POL317_F | GTTTGCCAGGTTCGTTTTGT | 59 | 536 |
|  |  | POL317_R | TCTAATCAAGTGGGGCAACC |  |  |
|  |  | POL318_F | CCTTAGTGGATCCATTGGAAGA | 59 | 686 |
|  |  | POL318_R | TTTTCCCCTAAAGAATACAAGGTT |  |  |
|  |  | POL319_F | ATTCCCCTTCATGAGCACAG | 59 | 598 |
|  |  | POL319_R | CCAGACTTGGACACCTGGTT |  |  |
|  |  | POL320_F | TAATAAGCTGGGGGCCAAAT | 59 | 850 |
|  |  | POL320_R | TCTTAGCTTGAGCCAATAAGAAAA |  |  |
|  |  | POL274_F | GGCCTGTTGCTTGATGAAAT | 60 | 615 |
|  |  | POL274_R | TCTGACGTCTGCTCAGGCTA |  |  |
|  |  | POL321_F | GGGTCCTAAGTGATGACATTGA | 59 | 640 |
|  |  | POL321_R | CAACCTTCCTCCCCAATTCT |  |  |
|  |  | POL322_F | CCTGGGTGTAGCAGCCATAG | 59 | 679 |
|  |  | POL322_R | TCTTACATAATCCAGCTTCACACC |  |  |
|  |  | POL323_F | GCCTTGTTGCAGCAATTAAA | 59 | 729 |
|  |  | POL323_R | TCCAGGTAGCCAAATCGAAC |  |  |
|  |  | POL324_F | TTTTGGAGGGAGGACCATAA | 59 | 608 |
|  |  | POL324_R | ACCCTACAGAATGGCCTGTG |  |  |
|  |  | POL325_F | TCAGTGTCGCCTATACCTCTG | 59 | 669 |
|  |  | POL325_R | ACCCATGGCAGGCTTCTTAT |  |  |
|  |  | POL326_F | GTAACTCCGCGTGTGGAAAG | 59 | 677 |
|  |  | POL326_R | CACAACAGGCAAAAGAATCC |  |  |
|  |  | POL327_F | GCAGAATTCGATCCAGGTGT | 59 | 627 |
|  |  | POL327_R | CTCCTTTGCCTTATCCCACA |  |  |
|  |  | POL328_F | GCTTTGGTGGAGTTGAGAGG | 59 | 626 |
|  |  | POL328_R | AGCAGCAGCACTGAGAAACA |  |  |
|  |  | POL329_F | TGCTTCAGTTGATGGATTGC | 59 | 633 |
|  |  | POL329_R | GATGGACTTGGTAAGTCAAACCTT |  |  |
|  |  | POL330_F | CAGTGTACCCTTCATTGTCTGC | 59 | 624 |
|  |  | POL330_R | AAAGGAACTGAGGCGCAAC |  |  |
|  | 3’flanking region | POLJ046_F | GGGGGAGACAGCATCAGATA | 60 | 694 |
|  |  | POLJ046_R | CCCAACAACCAAGAAGAGGA |  |  |
| intergenic |  | POLJ044_F | AGACTTCAGGCAGCGATTTG | 60 | 619 |
|  |  | POLJ044_R | TTCCTCGATGGCTACTGGAC |  |  |
|  |  | POLJ045_F | CTTTATTGTCCGTGCCCACT | 60 | 671 |
|  |  | POLJ045_R | TTCAGCTGATGTTGCCTTTG |  |  |
| *ATP5O* | Exon1 | POL120_F | CACAACAAATGCCACCCTAC | 58 | 591 |
|  |  | POL120_R | AGGACGCCAAGGTTAGAAAC |  |  |
|  | Intron1 | ATP5O_2_F | TGGGTCAACAGTAATCCTAAAGAA | 60 | 485 |
|  |  | ATP5O_2_R | AACAAGTGGGACGATTCCAG |  |  |
|  |  | ATP5O_3_F | TCTTGGTAGGAAACAAGCTAGAGAA | 60 | 529 |
|  |  | ATP5O_3_R | AGGCTGAGCTGCTCTTCATC |  |  |
|  | Exon2 | POL121_F | GTTTCCTCTCCCTTACTCGTG | 58 | 634 |
|  |  | POL121_R | TGTGTGATTGTACTGGAAAAGC |  |  |
|  | Exon3 | POL122_F | ACCTGTTTTCCGGCTTTAAC | 58 | 720 |
|  |  | POL122_R | CCCAAATCTCGGTTCCTAAC |  |  |
|  | Intron3 | ATP5O_6_F | CAGGTTCCCTGAGTCGTCTG | 60 | 560 |
|  |  | ATP5O_6_R | CAGCATCACGTTTTCCTCCT |  |  |
|  |  | ATP5O_8_F | TCCCGGACTTGACGTTTGT | 60 | 572 |
|  |  | ATP5O_8_R | GCTGCTGTACACACGCTTTC |  |  |
|  | Exon4 | POLJ043_F | ATCAGGTTGGACGTGAGAGG | 60 | 677 |
|  |  | POLJ043_R | GAGTCGCCTCTCTTGTGGTC |  |  |
|  |  | POL123_F | TCTGTGGTGCATTCTTAAAGG | 58 | 507 |
|  |  | POL123_R | GGGTGAAACTACGCATTGAG |  |  |
|  | Intron4 | ATP5O_9_F | CCTCTCACGTCCAACCTGAT | 60 | 599 |
|  |  | ATP5O_9_R | GGATGTGGATGGAGAAGCTG |  |  |
|  | Exon5 | POL124_F | TGTTAATTTGGGGATTCGTG | 58 | 919 |
|  |  | POL124_R | GCTTCATCACCACAAAAGATG |  |  |
|  | Exon6/ Exon7 | POL125_F | AGTGGGTCCCCAGATACC | 58 | 846 |
|  |  | POL125_R | TGAAGGCTATTTTCCCCTAAG |  |  |
| *ITSN1* | Exon1 | POL172_F | TGAGTCCTTTCTTCCGATTG | 58 | 573 |
|  |  | POL172_R | GAGGATGGGAAGGACGAG |  |  |
|  | Intron1 | POLJ034_F | GGCACATGGTACCTTCACAA | 60 | 697 |
|  |  | POLJ034_R | AACATGCCATCCTCTTCAGG |  |  |
|  |  | POL031_F | AACACGCACGTGTGTAAAATAG | 58 | 765 |
|  |  | POL031_R | TTTTTCAGAAGGGCACAGG |  |  |
|  |  | POLJ027_F | CTTCTGATTCCCACCCCACT | 60 | 383 |
|  |  | POLJ027_R | TGATGGCATTGTGAAGAACTG |  |  |
|  |  | POLJ041_F | ATATCCCTTTGGTGCTGACG | 60 | 544 |
|  |  | POLJ041_R | GTCAGTCAGGGCCTCACAAT |  |  |
|  |  | B360SP6_F | TCATTAGCACTGCCAGTTCTTC | 59 | 348 |
|  |  | B360SP6_R | CCAGGAGAGGGCAAATTCAAC |  |  |
|  |  | POL029_F | AGAGTCGGACACAACTGAGC | 58 | 647 |
|  |  | POL029_R | AGTGGCTTCTCTAAGGTTTTTC |  |  |
|  |  | POL002_F | CCCCCATATCTCTTACTTTTGG | 58 | 518 |
|  |  | POL002_R | GTCTCTTGAACTCAGTTCTCTC |  |  |
|  | Exon2 | POL173_F | TCCACTAAAATGTCTGTGGATG | 58 | 482 |
|  |  | POL173_R | CCGGGACTAAGAAGCTGTG |  |  |
|  | Exon3 | POLJ021_F | CTCCCCCATCTCTGTTTTGA | 60 | 489 |
|  |  | POLJ021_R | GCAAACCCCAACAAACAAAC |  |  |
|  | Intron3 | POL001_F | AGGCAAAGTACAAAGGCATC | 58 | 644 |
|  |  | POL001_R | CTTCCAAGGACAAACAGATG |  |  |
|  | Exon4 | POL174_F | CCAAGAAATGTGGGACACTC | 58 | 554 |
|  |  | POL174_R | TTACGTTCTAAAGTCCATGCTG |  |  |
|  | Exon5 | POL175_F | TGCATGCGTATAAGTGAAGAC | 57 | 531 |
|  |  | POL175_R | CCCTTCCAGCATTATCTTTG |  |  |
|  | Exon6 | POL176_F | TGCTCTTGGTTTTAGCTGTTG | 57 | 500 |
|  |  | POL176_R | GCATTCCTGGGTTAATTATTG |  |  |
|  | Exon8 | POL178_F | TGAGGCAGTGGAGAATGAAG | 58 | 498 |
|  |  | POL178_R | CTGTATGCAGAGAAAGGGTTG |  |  |
|  | Intron8 | B359T7_F | ATCGGTGAACTTTCCTCATTCC | 59 | 358 |
|  |  | B359T7_R | GCCAATATCACAGCCATTTCC |  |  |
|  | Exon9 | POL179_F | AGAGGGTAGTAAAAGGGACAAC | 57 | 372 |
|  |  | POL179_R | AAGCACTGTGATTGCATGAG |  |  |
|  | Exon10 | POL180_F | CCAGCTAGATGGGTTGTCTC | 58 | 454 |
|  |  | POL180_R | CGTGGAGATTTCATTCACTTG |  |  |
|  | Exon12 | POL006_F | GCCTCTCGATTTCCTTCCTTC | 58 | 720 |
|  |  | POL006_R | CAGGAGCTAGGCTTCAATTGC |  |  |
|  |  | POL182_F | CTCATTGACGATGTGCTGTG | 58 | 391 |
|  |  | POL182_R | CCCGTGTTAATGACATGACTG |  |  |
|  | Exon13/ Exon14 | POL183_F | CTTCATCTTGGCCTGCTG | 57 | 495 |
|  |  | POL183_R | CGTCTGTTTGTGTATGTCACAG |  |  |
|  | Exon15/ Exon16 | POL028_F | GCTGTAGTCGATTTGGGATG | 58 | 1809 |
|  |  | POL028_R | GATGAAGATGTGCAAAAATGC |  |  |
|  | Intron16 | POL030_F | TTGCTCCTAGGAACTGAATTG | 58 | 502 |
|  |  | POL030_R | TTGAAGGAATCTGCCTGAAG |  |  |
|  |  | POLJ022_F | GGAAAGGTGGGTCGTTGTTA | 60 | 689 |
|  |  | POLJ022_R | ACGGCCCATTTCTATACTGG |  |  |
|  | Intron17 | POL003_F | GAGGAGGAGAAGCTCAAAAGG | 58 | 594 |
|  |  | POL003_R | AGTGCAGACCAAGAACAGTG |  |  |
|  | Exon18 | POL007_F | TGTCATCTCTTCTCACAGATGG | 58 | 710 |
|  |  | POL007_R | CCCCTGTTTTGTTGATTCAG |  |  |
|  | Exon19 | POL185_F | CAGGAACCCATTTTTCTTTAAC | 57 | 462 |
|  |  | POL185_R | TGAAGACAGACAGCAGAGGAG |  |  |
|  | Exon20 | POL186_F | CGGAGCCTTCAAGTTAGTCC | 59 | 358 |
|  |  | POL186_R | CAGCCCCTCTGAAAGAATAAAC |  |  |
|  | Exon21 | POL187_F | AAAAAGGCAGAGAAAGGTCTG | 58 | 545 |
|  |  | POL187_R | TCCAGCTCAGAGAATACAATCC |  |  |
|  | Exon22 | POL188_F | TCCTTGCTCTTGAACTAGCC | 57 | 348 |
|  |  | POL188_R | GACAATGACGACAAGCACTG |  |  |
|  | Intron25 | B343T7_F | GTTCCTATCAGCTCCGATTCG | 59 | 410 |
|  |  | B343T7_R | GGCTATGGGTCTGGTAAATGG |  |  |
|  |  | B360T7_F | TCATTAGCACTGCCAGTTCTTC | 59 | 348 |
|  |  | B360T7_R | CCAGGAGAGGGCAAATTCAAC |  |  |
|  |  | B326SP6_F | TAAACTAGCTGAGCAAGCCAAG | 59 | 520 |
|  |  | B326SP6_R | GCACATTAAAGTGGCTGGAAC |  |  |
|  | Exon27 | POL191_F | CAAATGCAGGATTCAGACAG | 57 | 444 |
|  |  | POL191_R | AGAAGGCGAGTCCAGTCC |  |  |
|  | Exon28 | POL192_F | AAAGACAACTTTCGCCACTC | 58 | 292 |
|  |  | POL192_R | AGCACACCATGTTCAGAAGC |  |  |
|  | Intron29 | POLJ042_F | GTTGACACAGGTTTGTCTCCAG | 60 | 601 |
|  |  | POLJ042_R | GACCAGTTACCACTTGGTAGGG |  |  |
|  | Exon31 | POL201_F | TGTAAACCTGTGCGTGGAC | 58 | 477 |
|  |  | POL201_R | GTGCCTGTGAGCAGAAATG |  |  |
|  | Exon32 | POL193_F | CAGGGACACCTCTCTGTAGC | 58 | 485 |
|  |  | POL193_R | TCCTGTGCAGCTGAGACC |  |  |
|  | Exon33 | POL194_F | CTCCCCAGTTGTCATCCAG | 58 | 373 |
|  |  | POL194_R | ATGCAGACACCTCCTACAGC |  |  |
|  | Exon34 | POL195_F | CGTGTCATTGCCAAGGTG | 59 | 463 |
|  |  | POL195_R | GATCAGATGCAGCTGGAGAG |  |  |
|  | Intron35 | POL012_F | GCTGTCTTTGGCATTCTTGTC | 58 | 439 |
|  |  | POL012_R | CATGTTGCTGGCTACCTGTC |  |  |
|  | Exon35 | POL196_F | AAGTCTTCAAAGCCGTCCTC | 59 | 499 |
|  |  | POL196_R | AATCCTGCCTCACCTCCTC |  |  |
|  | Exon36 | POL197_F | AGACACAAACGGACACACC | 57 | 348 |
|  |  | POL197_R | GCAAGTGAGGGGATGTTG |  |  |
|  | Exon37 | POL198_F | GACAGCAACAGCGTTTATCAC | 58 | 494 |
|  |  | POL198_R | TCACCCCATACCCTACCC |  |  |
|  | Exon39 | POL202_F | GCATCTTAAGCATGGCATTG | 58 | 499 |
|  |  | POL202_R | TGGACCACTCTAGACCAAGC |  |  |
|  | 3’flanking region | POL025_F | GTGAGTACAAGGGCACTCAAG | 58 | 694 |
|  |  | POL025_R | GGAAAGGGCAACAAAGTTTAC |  |  |
| *CRYZL1* | Exon1 | POLJ033_F | AGTATCTCCCTCCCCTCAGC | 60 | 661 |
|  |  | POLJ033_R | GCACAAGTTAGGACGCCTTC |  |  |
|  | Exon2 | POL077_F | AGATGCTTCAGGATGTCCAC | 58 | 1038 |
|  |  | POL077_R | GGCTGCAATCTAAAAACTCG |  |  |
|  | Exon3 | POL078_F | TTTTACCCCGAATTCCCTAC | 58 | 525 |
|  |  | POL078_R | TCCAAATGATTGCTTGTGG |  |  |
|  | Exon4 | POL078_2_F | AACGAATCCCTTAGCAAACC | 58 | 838 |
|  |  | POL078_2_R | TAGGTTCCTTTGTGGAGCAG |  |  |
|  | Exon5 | POL016_F | TCTCATTCTCTTCATCAATGG | 58 | 617 |
|  |  | POL016_R | GCACTTCTGCAGCTAAAACC |  |  |
|  | Exon6 | POL079_F | AACCAAGGTTAGCCGTACATC | 58 | 532 |
|  |  | POL079_R | ATGACGACCACCCAATAGC |  |  |
|  | Exon7 | POL080_F | TGAAGGCCTGTGTTTTCTTC | 58 | 737 |
|  |  | POL080_R | TAAGTGGATGGCCCATTTC |  |  |
|  | Exon8 | POL081_F | ACAGGTCAGCAAAAGAATGG | 58 | 816 |
|  |  | POL081_R | TGCTAGGGGTCTAGTGCTTG |  |  |
|  | Exon9 | POL082_F | GTTTTGGAGGGCAAGTCAG | 58 | 792 |
|  |  | POL082_R | CCTGATGGTGCTTAAAATGG |  |  |
|  | Exon10 | POL083_F | GGTACGATTGAAACGGTCAG | 58 | 925 |
|  |  | POL083_R | CATCGAAGACTGGAACATGAG |  |  |
|  | Exon11 | POL084_F | CATCCTCATGAAAATGACTGG | 58 | 967 |
|  |  | POL084_R | TGACCTGTGATTTTCTTGAGG |  |  |
|  | Exon12 | POL085_F | ACCCCATTTTCCTGGTTTAG | 58 | 271 |
|  |  | POL085_R | TGAAACAAAAATGGGAATGC |  |  |
|  | Exon13 | POL086_F | CTTCGCTTATCCACATCACC | 58 | 977 |
|  |  | POL086_R | AGCCATTCACGCAAACAC |  |  |
|  |  | POL022_F | CCTCAGACCTCAGTTGGATG | 58 | 422 |
|  |  | POL022_R | GTGTGGATTTCCGATGACAG |  |  |
| *DONSON* | Exon1 | POL060_F | ATTCACTCCAGGGTTTCCTC | 59 | 1192 |
|  |  | POL060_R | TGTATTCTTCCCCTCAAACG |  |  |
|  | Exon2 | POL015_F | TGATGCGTTGGACTTGTTAC | 58 | 455 |
|  |  | POL015_R | TGGGCTACCTGAGAAACTTG |  |  |
|  | Exon3 | POL059_F | TGGAAGGAAATATCGCTGAC | 58 | 465 |
|  |  | POL059_R | CCTTCTGGACGTTTTCACAG |  |  |
|  | Exon4 | POL058_F | CAGAAGGACAGAGAAATCCATC | 58 | 469 |
|  |  | POL058_R | GACTGTAATTGCTGGCAAGG |  |  |
|  | Exon5 | POL057_F | TTCAGATTGGGACTTTCCAG | 58 | 602 |
|  |  | POL057_R | TGAGTCCCATGACCAATTTC |  |  |
|  | Exon6/ Exon7 | POL056_F | TCCCCAAATTTACCCAAAAC | 58 | 796 |
|  |  | POL056_R | GAAAGCATGGAAAACACTGC |  |  |
|  | Exon8 | POL055_F | AAAGGGGAAAATATGTCTTCAG | 58 | 366 |
|  |  | POL055_R | AGTAGGGAAAAGGGTTCCTG |  |  |
|  |  | POL024_F | GTCGGACGTAACTGAGCAAC | 58 | 854 |
|  |  | POL024_R | TGTGGCTTGCTATTTCTTCC |  |  |
|  | Exon9 | POL054_F | CCATGAATATAGTTGGGGATTC | 58 | 583 |
|  |  | POL054_R | GGAATGCCCAAATGAGAAG |  |  |
|  | Exon10 | POL061_F | AAATTAGCATTTGGGGATGC | 58 | 1072 |
|  |  | POL061_R | GCCCAAATATAAACTTCTCTGG |  |  |
| *SON* | Exon1 | POL036_F | TGCGTCAGTCACTAACATGG | 58 | 552 |
|  |  | POL036_R | AGAGAGCCTGGGATTACCTC |  |  |
|  | Exon2 | POL037_F | GGGAAAAGGGGAATATCATC | 58 | 567 |
|  |  | POL037_R | TTCAAAATCCTGGCACTGAG |  |  |
|  | Exon3 | POL038_F | TTTTTAACCCTGGCTTTAGTTG | 58 | 815 |
|  |  | POL038_R | ATGATCTTTGATGGCTCTGG |  |  |
|  |  | POL039_F | CAGTAATGCTGGAACCATCC | 58 | 823 |
|  |  | POL039_R | TCGGTCAACTCCATTGCTAC |  |  |
|  |  | POL040_F | CCCTTTCTACCCCAGTGC | 58 | 917 |
|  |  | POL040_R | ATGGGTCTCCATGGTGTTAG |  |  |
|  |  | POL041_F | ACAACATCGGAGCTGTCAAC | 58 | 1014 |
|  |  | POL041_R | AACGTTCTGCAGCATAGGAC |  |  |
|  |  | POL042_F | TTAGGCCATGATCCCTACAG | 58 | 977 |
|  |  | POL042_R | ACCATTTCATGCTCTTCTGC |  |  |
|  |  | POL043_F | AGAGGCTGACGTGACTGTTC | 58 | 972 |
|  |  | POL043_R | TCACCCATTTCACCAATAGC |  |  |
|  |  | POL044_F | GATAGCCATGCATTCAGAGG | 58 | 1027 |
|  |  | POL044_R | TGCCCTAGAACGAGATTCAC |  |  |
|  |  | POL045_F | GCTTCAGAGTCGTCTTCAGAG | 58 | 922 |
|  |  | POL045_R | CCCTCAAAAACCAATAATCC |  |  |
|  | Intron3 | POL046_F | TCAGAAAGAGGCAGATCACC | 58 | 910 |
|  |  | POL046_R | CATTCCTCTCCATTGTGCTC |  |  |
|  | Exon4 | POL047_F | GAGTGGGAAGCTAAATACGG | 58 | 872 |
|  |  | POL047_R | TGGTACCAAACAGCCAAAAC |  |  |
|  | Exon5/ Exon6 | POL048_F | CTGACTGACCAGCTAGAGTGTG | 58 | 766 |
|  |  | POL048_R | GAGTGAAACAGAATCCAAATCAG |  |  |
|  | Intron6 | POL014_F | CTCCTGGAAATTTCAAGGTGTG | 58 | 1425 |
|  |  | POL014_R | TGACTGACCAGCTAGAGTGTG |  |  |
|  | Exon7 | POL049_F | GCAACAAAAGATGTGAGCAG | 58 | 269 |
|  |  | POL049_R | TCAACAGCATGCCAGTTTAC |  |  |
|  | Exon8 | POL050_F | TTTGTGGGAGCCAAATAAAC | 58 | 454 |
|  |  | POL050_R | ACAAAACCCAGGAGCAGTAG |  |  |
|  | Exon9 | POL051_F | AAATTTTTGACTCCCTCTGTTG | 58 | 1033 |
|  |  | POL051_R | TGTAACTTTGTAAAGAAAGCAAGC |  |  |
|  | Intron9 | POL021_F | AGTGGACGAAAACAGGAGTG | 58 | 560 |
|  |  | POL021_R | TAAGAAGTGCCCCAGTTCAG |  |  |
|  | Exon10/ Exon11 | POL052_F | TTATTTGAGGGCATGGATTG | 58 | 604 |
|  |  | POL052_R | TCTCCAGATTGTACCCACATC |  |  |
|  | Exon13 | POL053_F | ATTTGCTTCTTTTGGGGTTG | 58 | 1130 |
|  |  | POL053_R | CTGGGTGCCGATTTTATTAG |  |  |
| *GART* | Intron4 | POL020_F | CCTGCCTCCTGTACAATGTC | 58 | 576 |
|  |  | POL020_R | GCACTGTGAAGACAGAGGAAG |  |  |
|  |  | POL065_F | TGGTATGCTCTCAGGGTAGG | 58 | 659 |
|  |  | POL065_R | GCAGTGAGACCCAGCACA |  |  |
|  | Intron5 | POL066_F | GAACCTGGAAAGGAGGCTAC | 58 | 1092 |
|  |  | POL066_R | CCAAAGTGAGGCCATAAAAG |  |  |
|  | Intron15 | POL071_F | TGTTTGCAGAATACAGCTTCC | 58 | 956 |
|  |  | POL071_R | CCACAAGAAGTCCAAATGATG |  |  |
|  | Exon17 | POL072_F | CAGATGGATCCCCAGAAAG | 58 | 533 |
|  |  | POL072_R | TAGACCCTGAGGCAGAAATG |  |  |
|  | Exon19 | POL074_F | GCTTGTTCCTCATCCTTCAG | 58 | 934 |
|  |  | POL074_R | ATCACTGGGTCATTCTCTGG |  |  |
| *LOC784171* | Exon2 | LOCF2_F | CCCCAGAATTTCCTTTAGGC | 60 | 747 |
|  |  | LOCF2_R | TGTGCTGGTACAATCATTTGC |  |  |
|  | Exon3 | POL288_F | GCACTTCCGGGAGAACTACA | 60 | 508 |
|  |  | POL288_R | GTTGGGATCGAGGACTTTCA |  |  |
|  | Intron3 | POL309_F | TGAAAGTCCTCGATCCCAAC | 60 | 537 |
|  |  | POL309_R | GAAAATGCAGACCCAGAAGG |  |  |
|  | Intron3 | POL289_F | TTTTTCGACTTGGAGCGAAT | 60 | 542 |
|  |  | POL289_F | CGCATACAATAAGGGCAGTTC |  |  |
|  | Exon4 | LOCFC_F | CCAGATTCAGCAGCCCATAA | 60 | 745 |
|  |  | LOCFC_R | GGGGTTAGTTAGTGAGGAGTAGAA |  |  |
|  | Exon4/ 3’UTR | POL161_F | CCTGTACTGCTTCTCTCGTTG | 57 | 595 |
|  |  | POL161_R | GCCCTTATTGTATGCGTAATC |  |  |
|  |  | POL162_F | GCCTAGTCAGGAGGGGAAC | 58 | 650 |
|  |  | POL162_R | ACAGACAAATGCCAGACAGAG |  |  |
|  |  | POL210_F | TCAGTTAGCTTGGCTGATGG | 59 | 782 |
|  |  | POL210_R | CCGAACAGAAACAGTGGAAC |  |  |
| intergenic |  | POLJ010_F | ACTGCGGCCTGTAGAGATGT | 60 | 522 |
|  |  | POLJ010_R | GGTGACTTTCTTGGCTGGAA |  |  |
|  |  | POLJ016_F | TCCAATTGTAGCATCCGTGA | 60 | 625 |
|  |  | POLJ016_R | GCTCCATCTGTCCCCATAAA |  |  |
| *TMEM50B* | Intron1 | POL294_F | GCCCAGATGAGGTCCTAACA | 60 | 683 |
|  |  | POL294_R | CACACAGGAGAGCTGACGAG |  |  |
|  |  | POL295_F | GAGGATGGAGTGGAGCTGTC | 60 | 595 |
|  |  | POL295_R | CTCCCTTCCTTCCTTCCTTG |  |  |
|  | Exon3 | POL292_F | GGCCCATAGAAAACGTGAAA | 60 | 670 |
|  |  | POL292_R | TTCAAGGATTACTCCCCTCCT |  |  |
|  | Exon4 | POL127_F | CCTGGAGTCAAGATCCACTG | 58 | 627 |
|  |  | POL127_R | CTCATGTAAAATCGGCAAGG |  |  |
|  | Intron4 | POLJ028_F | CATTTGTGCCAGACCCTGAT | 60 | 626 |
|  |  | POLJ028_R | GGAACCCCAGGTAGAAAGGA |  |  |
|  | Exon5 | POL019_F | GGGTAACATAGGCACCAAAG | 58 | 517 |
|  |  | POL019_R | TCATTTGCAGTCTCTCTTTCC |  |  |
|  | Exon6 | POL293_F | TGTGGATTGCTTGGAGTTAATG | 60 | 600 |
|  |  | POL293_R | AACATGGATAAATCTACAGTGG |  |  |
|  | Intron6 | POL215_F | AGGCACAGCAAACAGAGGAG | 60 | 531 |
|  |  | POL215_R | CTGTCAGTACTCGGCAGAAGC |  |  |
|  |  | POL214_F | ACTGGGAATGGGACTGTGAG | 60 | 540 |
|  |  | POL214_R | TTTCTGGCCTTTGCTTCTTC |  |  |
|  | Exon7 | POL207_F | TGCACATCTTTGAAAATCAGG | 58 | 724 |
|  |  | POL207_R | TGCTTCAGTTTTTGTAGCTCAG |  |  |
|  | Exon7/ 3’UTR | POL101_F | AAAAACAAAGGAAAGCCAAAG | 58 | 800 |
|  |  | POL101_R | TGAGCCAGGGTTGTATCATC |  |  |
|  |  | POLJ015_F | CGCGGTAAAACCTCAGAGAG | 60 | 619 |
|  |  | POLJ015_R | GGTAGGCAGCTCAGCAACAT |  |  |
|  |  | POL157_F | GGAGCAAGAATCTCACAAGC | 58 | 652 |
|  |  | POL157_R | TTGAAAGCTTCTTTTGCTGAG |  |  |
|  | 3’flanking region | POL158_F | AGCCTTCACACTAGCACCTC | 58 | 239 |
|  |  | POL158_R | TTACATGTTTCCACTCCCTTG |  |  |
| *IFNGR2* | 5’flanking region | POL386_F | AGCCAGGAGAAAGAGGAAGC | 60 | 751 |
|  |  | POL386_R | GCAGCGCACTATTCCGTAAG |  |  |
|  |  | POL312_F | TGTCCAAAAGCAAAGCTTGA | 60 | 359 |
|  |  | POL312_R | GCCCCACATTTCATAATGGT |  |  |
|  |  | POL219_F | TTATGCCTCCTTCCTTTCCTG | 61 | 597 |
|  |  | POL219_R | CGACCTTGGCTCAGAGGAG |  |  |
|  | 5’flanking region/ Exon1 | POL279_F | GAGGGGTCTTTCCCGAAG | 60 | 784 |
|  |  | POL279_R | GAACCCCTTCCACTGATCCT |  |  |
|  | Intron1 | POL220_F | CGTCTCGAGAGTGGGAGTC | 59 | 798 |
|  |  | POL220_R | GAGCTTAGGTGGGGCAGTC |  |  |
|  |  | POL376_F | CGGCTCGACTCTGAATGTCT | 60 | 757 |
|  |  | POL376_R | ATGGAGTTTAATGGCGTTG |  |  |
|  |  | POL221_F | TTGTGTTTTGCTGGCTTATTTC | 59 | 671 |
|  |  | POL221_R | CAAGACTCGAAGCTTGAGGAG |  |  |
|  |  | POL377_F | GCAACGCCATTAAACTCCAT | 60 | 903 |
|  |  | POL377_R | CTCTGGACCACAGCAGACAA |  |  |
|  |  | POL222_F | AGATGAGATTCGGGTGTTGG | 60 | 644 |
|  |  | POL222_R | AGACCCTTCAGGATCCTTCC |  |  |
|  |  | POL378_F | TCCAGTATTTACGGACAAGACC | 58 | 403 |
|  |  | POL378_R | ACATTTTTAAGAACAGGCTCTCC |  |  |
|  |  | POL223_F | AGGGGTCTTCGATTGTGAAG | 59 | 686 |
|  |  | POL223_R | TCAACTTGATAAAGCCATCAGC |  |  |
|  |  | POL379_F | TTGCCACAAGCATTTTCAAC | 60 | 798 |
|  |  | POL379_R | CAAGCATTTCCACTGCAGAC |  |  |
|  |  | POL380_F | TCAGTTGTTTGCACAGTATTGC | 59 | 793 |
|  |  | POL380_R | TCCAGTGACCACCAACAGAG |  |  |
|  |  | POL224_F | GCGAGGTCAGACCATTGAC | 59 | 641 |
|  |  | POL224_R | ACTTATCTGGATGGCTCTAAAGG |  |  |
|  |  | POLJ025_F | GCAGGTCATATGGCCCTAAA | 60 | 496 |
|  |  | POLJ025_R | ACGGAGCTGTAGCCCAGTAA |  |  |
|  |  | POL381_F | TGGACAAATGCAGAAACTGG | 60 | 498 |
|  |  | POL381_R | CAATCCCCCTTTCCTTTCTC |  |  |
|  |  | POL225_F | TGTAAACACAGGACCCAGGTG | 60 | 597 |
|  |  | POL225_R | CCCATCTAGACAGTCCGCTAAG |  |  |
|  |  | POL382_F | AGGGAGAAAGAAGGCTCCAG | 60 | 998 |
|  |  | POL382_R | AGGGTCTCAGGGGGTGAG |  |  |
|  |  | POL2ID_F | TGCACCTTTCATGTAACAGAGG | 58 | 168 |
|  |  | POL2ID_R | GCTGCTGGTGTGAGTGAGAG |  |  |
|  |  | POL226_F | CCTAACCAAGCCCTCACC | 58 | 600 |
|  |  | POL226_R | CTGCCAATGTCAGAATCTCC |  |  |
|  |  | POL383_F | CCTCACGAGAGAAAAGCCTTC | 60 | 417 |
|  |  | POL383_R | CTTGATGGCTACTGGACAAGC |  |  |
|  |  | POL227_F | CCAGGTGTCTTTGGGATTTG | 60 | 666 |
|  |  | POL227_R | CAACAAAAGATGGCACAGTTG |  |  |
|  |  | POL384_F | GAACTGGAACCTGCAGTTGG | 60 | 508 |
|  |  | POL384_R | GGAGATTGCATGAAGGGTTC |  |  |
|  | Exon2 | POL152_F | AGATAAGAAAGTTGAGGGAACC | 56 | 497 |
|  |  | POL152_R | GACGACTTTGCTTACAGTGC |  |  |
|  | Exon2/ Intron2 | POL362_F | ATGTGTGGGGAGAATGGGTA | 60 | 789 |
|  |  | POL362_R | CGGGCCAGAAGGCTAAATA |  |  |
|  | Intron2 | POL363_F | TTCATCAGTTTAAGGCGGAGA | 60 | 789 |
|  |  | POL363_R | CGTCAACTTCTAGCCAATGGA |  |  |
|  |  | POL364_F | AGTGGGAATGCCTAGTGCTG | 60 | 726 |
|  |  | POL364_R | CAATCCAGAGATAGGCCTCAA |  |  |
|  |  | POL365_F | GGAGGAGGTTTCCAGAGGAC | 60 | 796 |
|  |  | POL365_R | GCAGCGCTCTAGGAGACTGT |  |  |
|  |  | POL366_F | AGGCCTATCTCTGGATTGCAT | 60 | 723 |
|  |  | POL366_R | TTACCAAAACCAGCCCCTAA |  |  |
|  |  | POL367_F | GAGGGCCTCTGGACACTAGA | 60 | 735 |
|  |  | POL367_R | AGAGGATCTGCCACCTCAGA |  |  |
|  |  | POL368_F | GGAAGAGGGCCTTTGATTTT | 60 | 746 |
|  |  | POL368_R | CAGGACCCAGAGGTGTGACT |  |  |
|  |  | POL4ID_F | TGGGGTGTGACCTAGAAAGG | 58 | 198 |
|  |  | POL4ID_R | ATCTGAGGTGGCAGATCCTC |  |  |
|  |  | POL369_F | ATGCCAGCATCTGTGTGAGA | 60 | 427 |
|  |  | POL369_R | GGCAGCGGTGCCTACTAAG |  |  |
|  |  | POL370_F | CAGGGAAATCATTGGTTGCT | 60 | 728 |
|  |  | POL370_R | TTTGGCTCCCCATCTATTTG |  |  |
|  |  | POL371_F | CCATGAGGCTGTGTTCATTG | 60 | 752 |
|  |  | POL371_R | CTCCCCTCTCCTCCAAACTT |  |  |
|  |  | POL372_F | ACGGACAAGTTTGGAGGAGA | 59 | 701 |
|  |  | POL372_R | ATAGAGTCCTGCCCACAAGG |  |  |
|  |  | POL373_F | TACAGGCAGCAAGGTTTCCT | 60 | 455 |
|  |  | POL373_R | CCACACCCCAGTTTCTCACT |  |  |
|  |  | POL374_F | AGTGAGAAACTGGGGTGTGG | 60 | 1098 |
|  |  | POL374_R | ACTACTGGTGCTGCTGCAAA |  |  |
|  | Exon3/ Intron3 | POL097_F | GGGGGAACTGTGATCTGTG | 58 | 617 |
|  |  | POL097_R | AGGCTGTGGTTTAGCAACTC |  |  |
|  | Intron3 | POL244_F | AAGCGGTACTGACTCCATG | 59 | 993 |
|  |  | POL244_R | AGATGGGGAAACTGTGTTCTG |  |  |
|  |  | POL385_F | TGGAAAAGAATTTGAAAAAGAATG | 59 | 587 |
|  |  | POL385_R | CCAGATGATGGTGACCAGTG |  |  |
|  |  | POL245_F | TCAGAACACAGTTTCCCCATC | 59 | 474 |
|  |  | POL245_R | TGAGTTTGAGCCAACTCCAG |  |  |
|  |  | POL246_F | TGCCATCCAACCATCTTAGC | 60 | 683 |
|  |  | POL246_R | TCAGATGTCTGGCAGTGCTC |  |  |
|  |  | POL247_F | TTGAGGATGGGATGAGAAGG | 60 | 768 |
|  |  | POL247_R | AAGGGAGTGGGTGTATGTGC |  |  |
|  |  | POL248_F | TCACCGCACAGACCTATCAG | 60 | 680 |
|  |  | POL248_R | CGGAGGAACTCAGATCAAGG |  |  |
|  |  | POL249_F | CCTCCTCTCGTGAACAGGTG | 60 | 680 |
|  |  | POL249_R | AGGCTAGCTTTTATCAGGTTTCC |  |  |
|  |  | POL250_F | CTTCCAGCTCTCTGGAAACC | 59 | 398 |
|  |  | POL250_R | GTCACCCGGATGTTTTCTG |  |  |
|  | Exon4/ Intron4 | POL098_F | CACACATACATGCACACACG | 58 | 665 |
|  |  | POL098_R | GCATTGCCTTCTTTGAAAAC |  |  |
|  |  | POL098_mm1_F | GAAACTCCAAAGAAAAGAACATCAAACTCA | 62 | 195 |
|  |  | POL098_mm_R | CTACCATTGGGCCTCCAGA |  |  |
|  | Intron4 | POL313_F | CCGTGGTCTGAGTCTGGATT | 60 | 526 |
|  |  | POL313_R | ACCCCTCCTTTGTCAACCTT |  |  |
|  |  | B80SP6_F | GAATTCACCTTCCAATGCAGAG | 58 | 418 |
|  |  | B80SP6_R | AACGTAGGAAATCAGGAGGTTC |  |  |
|  |  | POL331_F | AGGGAGGTCCCTTCAAGCTA | 60 | 661 |
|  |  | POL331_R | CCAGACCTCCTCTGTTCCAG |  |  |
|  |  | POL332_F | AAGGGCAGACTCTCAGCAAA | 60 | 804 |
|  |  | POL332_R | AGCCAGTTGGCTATTCCAGA |  |  |
|  |  | POL333_F | AATAGCCAACTGGCTGCTGA | 60 | 667 |
|  |  | POL333_R | AATGGTGGCAGCTTCAGAGT |  |  |
|  |  | POL334_F | CTGTTGAATGATCTGCCTTTTT | 60 | 639 |
|  |  | POL334_R | GCTTCTGGTTTTCGCTTCAC |  |  |
|  |  | POL335_F | CCCAACTCTTCCTTGATGACA | 60 | 373 |
|  |  | POL335_R | AACGCTTACAGCCGACAAAT |  |  |
|  | Intron4/ Exon5 | POL099_F | TCTCACAGCCAGAAACACAG | 58 | 616 |
|  |  | POL099_R | GTTACCCGTCTGGCAGTTC |  |  |
|  | Intron5 | POL336_F | GATACAACGAGGCTGTGCTG | 59 | 475 |
|  |  | POL336_R | ACGTCCCAGGAAAATGAATG |  |  |
|  | Exon6 | POL206_F | TACAGGGCAGGGTGGAAG | 60 | 300 |
|  |  | POL206_R | GGGCGTGTACATACCTCCTC |  |  |
|  | Exon6/ Intron6 | POL126_F | AGGGTGGAAGAGAGCAGTATC | 58 | 607 |
|  |  | POL126_R | GGTGAATCACGAGGCTTG |  |  |
|  | Intron6 | POL337_F | CCTGCAAGCCTCGTGATT | 60 | 698 |
|  |  | POL337_R | CCATCTTTTGGCCAACCTC |  |  |
|  |  | POL338_F | GAGGTTGGCCAAAAGATGG | 60 | 722 |
|  |  | POL338_R | CTGGGCAGGAAAACAAGACT |  |  |
|  |  | POL340_F | ACAACAGCAACCGATTTTCC | 60 | 554 |
|  |  | POL340_R | ACGACCACCACAACCAAGTT |  |  |
|  |  | POL341_F | GCGCTAAGCTGACTGCATTT | 60 | 689 |
|  |  | POL341_R | CCAACCAAGGGGATACTCTG |  |  |
|  | Intron6/ Exon7 | POL263_F | CCAGTGCAGCAAGTCAGAAC | 60 | 533 |
|  |  | POL263_R | ACCCCCAAGGTCTTTTTGTG |  |  |
|  | Exon7/ 3’UTR | POL100_F | GGATGACACTTGGGACTCTG | 58 | 778 |
|  |  | POL100_R | GCCACTGAGTGTCCTTTGAC |  |  |
|  |  | POL342_F | TGACACCCTTGTGATATTTCG | 59 | 776 |
|  |  | POL342_R | GACGCATCCATTTCCTAAGC |  |  |
| intergenic |  | POLJ001_F | CAGAGGACACGGCCATATTT | 60 | 526 |
|  |  | POLJ001_R | CAGCCTGAATGTCAATGGTG |  |  |
|  |  | POLJ002_F | GGCTGAGAACCCTCATGTGT | 60 | 514 |
|  |  | POLJ002_R | TGGCAAGTCACTGTGAGGAG |  |  |
|  |  | POLJ003_F | TGGTTATGCAGGGAGCTTCT | 60 | 447 |
|  |  | POLJ003_R | TTGGGTAATGGCTCGGATAG |  |  |
|  |  | POLJ004_F | TGCCTCTTGTCTGTGGAAAA | 60 | 581 |
|  |  | POLJ004_R | CAGCTGCTCACAGCTCAAAG |  |  |
|  |  | POLJ005_F | CAGACGGCTTCGAGGAATAG | 60 | 522 |
|  |  | POLJ005_R | GGAGCACGTTCCACTAAAGC |  |  |
|  |  | POLJ006_F | CAACAAAACAGATGTCACCTCCT | 60 | 500 |
|  |  | POLJ006_R | GGCATTCAATCTGCTGGGTA |  |  |
|  |  | POLJ009_F | GGTTCAAAGGACCTGCACAT | 60 | 533 |
|  |  | POLJ009_R | GGTGGTGAGGATGCAAAGTT |  |  |
|  |  | POLJ026_F | TTCACACTGCGTGGCATAAT | 60 | 694 |
|  |  | POLJ026_R | CCTTGGAAATCAGACCTGGA |  |  |
|  |  | POLJ029_F | GTCCTCTCCAGAGCACCTTG | 60 | 749 |
|  |  | POLJ029_R | TGATTTCTGGCTGCAATGAC |  |  |
|  |  | POLJ030_F | AACCAAAGGACAGTGGTTGC | 60 | 654 |
|  |  | POLJ030_R | CCAAAACACTGGCCTGAAAT |  |  |
| *IFNAR1* | Exon1 | POL303_F | CGGTGTTGCAAGGCTTAGTA | 59 | 583 |
|  |  | POL303_R | AACCAGAGGTCTCCCAATGA |  |  |
|  | Exon3 | POL142_F | AATGTTCATTCATTCCTTTGC | 57 | 438 |
|  |  | POL142_R | TCTCTTGGAGACCACACTCTG |  |  |
|  | Exon4/ Exon5 | POL143_F | TTTTTGGCCACATTTCTCAG | 58 | 590 |
|  |  | POL143_R | TGAATTGAAAGCAAAACTTCC |  |  |
|  | Exon6 | POL144_F | TCTTGCCATCTGTTCTCTCAC | 58 | 271 |
|  |  | POL144_R | AGAACAACAGGTGGCTGAAG |  |  |
|  | Intron6 | B88T7_F | TTCTTCAAAACCCACTCCTTCC | 59 | 244 |
|  |  | B88T7_R | AAACTACACCCGGTCCTCTTC |  |  |
|  | Exon7/ Exon8 | POL145_F | GAGTTTCCCAAGGCTGAGTAG | 58 | 673 |
|  |  | POL145_R | TCCTCCCCAGGGAGAACAC |  |  |
|  | Exon9 | POL304_F | TTTGGTAATGGCGTATTGTCC | 59 | 557 |
|  |  | POL304_R | AGCATGCAGAAACCACTGAA |  |  |
|  | Exon10 | POL147_F | AACTTAGTTCAGTGGTTTCTGC | 56 | 610 |
|  |  | POL147_R | CTCTGTTACATGGCTGCTTC |  |  |
|  | Exon11 | POL305_F | CTGACCCCTCGTAGCACAGT | 60 | 559 |
|  |  | POL305_R | GGCTCCCACTCTGACTCTTG |  |  |
|  | 3’UTR | POL149_F | CCTGCCTTGGATGAGAGG | 58 | 700 |
|  |  | POL149_R | CCTGTTCCCCATTTCTCTG |  |  |
|  |  | POL306_F | AGGCTTCCTGTTTCAGAACATT | 59 | 618 |
|  |  | POL306_R | CTGCAACGACTGAAGCTGAG |  |  |
| intergenic |  | POLJ008_F | TCCGAGTCCCTTCACTGTTC | 60 | 547 |
|  |  | POLJ008_R | CCCGGGTTATTGGCTCTAA |  |  |
|  |  | POLJ013_F | CTCTGGATCCTCGCCAGTAG | 60 | 693 |
|  |  | POLJ013_R | GGTCAAGCCATTTCCTTTGT |  |  |
|  |  | POLJ014_F | AATGGACATGGGTTTGGGTA | 60 | 760 |
|  |  | POLJ014_R | AGGCAGAACTGGAGTCATGG |  |  |
|  |  | POLJ023_F | TGGGTACACATGGCTCTCAG | 60 | 650 |
|  |  | POLJ023_R | AACCAGAAGTCCCACCATTG |  |  |
|  |  | POLJ024_F | AAGCAGCAGGACAGGAAGTC | 60 | 496 |
|  |  | POLJ024_R | GGGCAGGTTAAAACAATGGA |  |  |
|  |  | POLJ031_F | CGCCAGTAGGTAACCACGTT | 60 | 720 |
|  |  | POLJ031_R | GAGGCTAAGTGGGGAAGGAA |  |  |
|  |  | POLJ032_F | CCACTGGTCATCTGGGAAGT | 60 | 623 |
|  |  | POLJ032_R | TGGCCCATCCTTATCACATT |  |  |
| *IL10RB* | Exon1 | POL300_F | TCCCCTTTCACCTCCTTTTC | 60 | 599 |
|  |  | POL300_R | GGGCAGAATTCAGACTTTGG |  |  |
|  | Exon2 | POL212_F | CACTGCTAAAGCGTGTCTCC | 58 | 540 |
|  |  | POL212_R | GACACCCACGATCTATAGGC |  |  |
|  | Exon3 | POL115_F | AGAATTCCAAGGTCCAGTCC | 58 | 573 |
|  |  | POL115_R | TTTCTGCCTTATGCTGATCC |  |  |
|  | Exon4 | POL116_F | TAAAAGGGAGGTGGGAAGAG | 59 | 669 |
|  |  | POL116_R | GCTTCTGGAGCTCAAAGGAC |  |  |
|  | Exon5 | POL117_F | AATAAAGCAGTTTGCCAGGAC | 58 | 618 |
|  |  | POL117_R | CTCGGTGGAAGTCTCTAAAGC |  |  |
|  | Exon6 | POL118_F | GGATGGTAGGAAAGGGTGAG | 58 | 613 |
|  |  | POL118_R | AGGTCAGGGAAGGAACAAAC |  |  |
|  | Exon7 | POL119_F | CATTTTCCAGCCAGGAGTTC | 60 | 684 |
|  |  | POL119_R | TATCTCTGGTGGCCAACCTC |  |  |
|  | 3’UTR | POLJ007_F | GAGGTTGGCCACCAGAGATA | 60 | 480 |
|  |  | POLJ007_R | ACAGCCACACAGATGGAGTG |  |  |
| *IFNAR2* | Exon1 | POL307_F | GGTGTACACGCGGGTAAAGTA | 60 | 602 |
|  |  | POL307_R | GCAGGGGACAGTCAGTGG |  |  |
|  | Exon2 | POL111_F | AAATGATGGGACCAGATGC | 58 | 610 |
|  |  | POL111_R | ACATTGGGCAGCTCATACTG |  |  |
|  | Exon3 | POL104_F | ATTTTGGATCCACTGAGGAC | 58 | 601 |
|  |  | POL104_R | TTCAAACAAAATGGCAACAG |  |  |
|  | Exon4 | POL105_F | TTTACACGGTGCTGAGTTCC | 58 | 643 |
|  |  | POL105_R | ATTTTGCATCTGAGGCTTTG |  |  |
|  | Exon5 | POL106_F | TACTTCCCTTTGCTGTCTGC | 58 | 655 |
|  |  | POL106_R | CCTGAGTGACGGCCTTATAC |  |  |
|  | Exon6 | POL107_F | AGGATTAACCGCATTCAAAG | 58 | 838 |
|  |  | POL107_R | ACAGTCTGCCCACTCATCTC |  |  |
|  | Exon7 | POL308_F | TAGGGCTTGCCTCTAAACGA | 60 | 655 |
|  |  | POL308_R | TCATCCACACTGGCAAGTTC |  |  |
|  | Intron7 | POLJ017_F | GGCTCAGATCTTCACCGAGT | 60 | 682 |
|  |  | POLJ017_R | TCTGGTGGTGGAAGGGTTTA |  |  |
|  | Exon8 | POL108_F | ATAAACCCTTCCACCACCAG | 58 | 888 |
|  |  | POL108_R | TGGTTGAGTGCCAAAGATTC |  |  |
|  | Exon9 | POL112_F | TTCCCATCTCTGTGCAGTTAG | 58 | 610 |
|  |  | POL112_R | TCCAGCTCGACTGTCTCTTC |  |  |
|  |  | POL113_F | CCGAGGAGGACAGTGACTC | 58 | 598 |
|  |  | POL113_R | TTGCCTTGAACAGGTAAACAG |  |  |
|  | 3’UTR | POL109_F | CTGCACTTGCTCCTTTGG | 58 | 843 |
|  |  | POL109_R | TGCCAGCAGAACTACACATC |  |  |
| intergenic |  | POLJ040_F | CTGGAGAACCACACTGAGCA | 60 | 526 |
|  |  | POLJ040_R | CTAATCACAGGCCAGGTTGG |  |  |
| *HIST1H4C* | Exon1 | POL284_F | ATGTCTGGTCGTGGGAAGG | 60 | 320 |
|  |  | POL284_R | TGTTTTGCTCAACCACCAAA |  |  |
| *LOC782947** |  | POL285_F | TGGGAAACCCCTTTCTTTCT | 60 | 473 |
|  |  | POL285_R | CCTGTCCCAGTTGGTTCTGT |  |  |
| interngenic |  | POLJ018_F | CCTTGGGGATGAATGCTTTA | 60 | 593 |
|  |  | POLJ018_R | ATCTCCCAGTGGTGAGTTGC |  |  |
|  |  | POLJ019_F | TAAACAGTTCCTCCCGTCGT | 60 | 618 |
|  |  | POLJ019_R | AACTCATGCCCAACCATCTC |  |  |
|  |  | POLJ037_F | CAGGAGGCAAAACCAACAAT | 60 | 539 |
|  |  | POLJ037_R | TCAACGGTGGATTTCTAGCC |  |  |
|  |  | POLJ038_F | GGGAATGCTTAAGGACCACA | 60 | 733 |
|  |  | POLJ038_R | GGGATAAGAAACGGCTCCTC |  |  |
|  |  | POLJ039_F | TAAGCCCCCTTTGCACTCTA | 60 | 696 |
|  |  | POLJ039_R | GTGGCCTGGTTCGTTTCTAA |  |  |
| *OLIG1* | 5’flanking region/ Exon1 | POL405_F | ACCATCCCAACACCAGTCC | 58 | 871 |
|  |  | POL405_R | ATGTAGTTGCGGGCTAGCAG |  |  |
|  | Exon1 | POL132_F | GTTCCCAAGCCGGGTTTA | 61 | 533 |
|  |  | POL132_R | CCGAGTAGGGCAGGATGAC |  |  |
|  | Exon1/ Intron1 | POL401_F | CGTTCCCCCAGATGTATTATG | 58 | 760 |
|  |  | POL401_R | AGGTGCGGGAACTTGCAG |  |  |
|  |  | POL410_F | AGAGAGAGGAAGCGGATGC | 60 | 496 |
|  |  | POL410_R | GCCCTCACTTGGAGAACTGC |  |  |
|  | Exon1/ Exon2 | POL402_F | GTCGGGGCCTCCCTCTAC | 58 | 890 |
|  |  | POL402_R | CGACGCTCTGTCGAACATC |  |  |
|  | Intron1/ Exon2 | POL403_F | TGCGCCGTCTGCAAGTTC | 58 | 838 |
|  |  | POL403_R | AGGAATCTCGAGGTCCAAAC |  |  |
|  | Exon2 | POL302_F | GCGCAGTTCTCCAAGTGAG | 59 | 530 |
|  |  | POL302_R | CACTGACAGCGTGGTCTTTC |  |  |
|  |  | POL135_F | AGAGCGATGCGGACTTTAG | 58 | 555 |
|  |  | POL135_R | GGAAAAGGTGGTTGTTTTTAAG |  |  |
|  |  | POL404_F | TTCTCTTCAGCCAGAGGTCAC | 58 | 866 |
|  |  | POL404_R | AAGGCCCTTACATGTTTATTTTG |  |  |
|  |  | POL136_F | CGATGATGCGCTCTTAAATC | 58 | 627 |
|  |  | POL136_R | CAATCCATCAGGGTATCAGG |  |  |
| intergenic |  | POLJ035_F | TCCTGAGTAATGCCCAGGTC | 60 | 601 |
|  |  | POLJ035_R | CACAGATTGCACTGGGTGAC |  |  |
|  |  | POLJ036_F | GCAGCAACAAAACCAAGACA | 60 | 604 |
|  |  | POLJ036_R | GTCACTCTCCGCTGAGAACC |  |  |
| *OLIG2-like** | Exon1 | POL296_F | TGACAGTGTCCCCTCTCTCC | 60 | 598 |
|  |  | POL296_R | GGGATCTGGATCCACTTTAGC |  |  |
|  | Exon2 | POL297_F | TCCATCCTCCAGGGATTAAA | 60 | 588 |
|  |  | POL297_R | AGAGGTGCTGGACGAGGATA |  |  |
|  | 3’flanking region | POL299_F | GGGAATTCAGTCCCAAACAG | 60 | 578 |
|  |  | POL299_R | AGAAGCGACAGGGAGAAACA |  |  |
|  |  | POL103_F | AAAGGAGTTACGGGTTAGGC | 58 | 702 |
|  |  | POL103_R | TGGGTTATTTGGGAGTTCTG |  |  |

* pseudogene
