# Supplementary material for: A Single Nucleotide Polymorphism within the Interferon Gamma Receptor 2 Gene Perfectly Coincides with Polledness in Holstein Cattle
Source: PLoS One. 2013 Jun 21;8(6):e67992. doi: 10.1371/journal.pone.0067992 (PMC3689702; doi:10.1371/journal.pone.0067992)
Supplement: Table S2 — (DOC) [file pone.0067992.s005.doc]

**Table S2. Polymorphisms located in the bovine *interferon gamma receptor 2* (*IFNGR2*) gene on BTA1 in Holsteins.**

| Polymorphism-ID | Localisation within *IFNGR2* (assembly UMD3.1) |
| --- | --- |
| AC000158:g.1376520T>C | Exon7 (UTR) |
| AC000158:g.1376880T>A | Exon7 (CDS) |
| AC000158:g.1376884T>C | Exon7 (CDS) |
| AC000158:g.1376932A>G~ | Exon7 (CDS) |
| AC000158:g.1376977A>G | Exon7 (CDS) |
| AC000158:g.1380674A>C~ | Intron6 |
| AC000158:g.1381425T>C | Intron5 |
| AC000158:g.1381634C>G | Intron5 |
| AC000158:g.1382090C>T | Intron4 |
| AC000158:g.1382184G>A | Intron4 |
| AC000158:g.1382414C>T* | Intron4 |
| AC000158:g.1382438A>G* | Intron4 |
| AC000158:g.1383255A>G | Intron4 |
| AC000158:g.1383518G>A | Intron4 |
| AC000158:g.1383725T>C~ | Intron4 |
| AC000158:g.1384105C>T | Intron4 |
| AC000158:g.1384140C>G | Intron4 |
| AC000158:g.1384180T>C | Intron4 |
| AC000158:g.1384465G>A | Intron4 |
| AC000158:g.1384673G>C | Intron4 |
| AC000158:g.1384726T>C | Intron4 |
| AC000158:g.1385009_035delinsATACAG | Intron4 |
| AC000158:g.1385271A>G | Intron4 |
| AC000158:g.1385373A>Y | Intron4 |
| AC000158:g.1385405C>T | Intron4 |
| AC000158:g.1385465G>A~ | Intron4 |
| AC000158:g.1385553G>A | Intron4 |
| AC000158:g.1385605C>A | Intron4 |
| AC000158:g.1385676G>C | Intron4 |
| AC000158:g.1385697G>A | Intron4 |
| AC000158:g.1385717G>A | Intron4 |
| AC000158:g.1385720G>A | Intron4 |
| AC000158:g.1385725G>A | Intron4 |
| AC000158:g.1385749C>T | Intron4 |
| AC000158:g.1385867C>T~ | Intron4 |
| AC000158:g.1385927G>A~ | Intron4 |
| AC000158:g.1386366G>T | Intron3 |
| AC000158:g.1386712A>G | Intron3 |
| AC000158:g.1386893C>T | Intron3 |
| AC000158:g.1387153delG~ | Intron3 |
| AC000158:g.1387428A>G | Intron3 |
| AC000158:g.1388179A>G* | Intron3 |
| AC000158:g.1388250A>G* | Intron3 |
| AC000158:g.1388281delT* | Intron3 |
| AC000158:g.1389833C>T* | Intron3 |
| AC000158:g.1390179C>A* | Intron3 |
| AC000158:g.1390268T>G | Intron3 |
| AC000158:g.1390292G>A | Intron3 |
| AC000158:g.1390505T>C | Exon3 (CDS) |
| AC000158:g.1390528T>C | Exon3 (CDS) |
| AC000158:g.1391644C>T* | Intron2 |
| AC000158:g.1392038A>T* | Intron2 |
| AC000158:g.1392215C>A* | Intron2 |
| AC000158:g.1393021insT* | Intron2 |
| AC000158:g.1394340A>G* | Intron2 |
| AC000158:g.1394768_771delCTGC* | Intron2 |
| AC000158:g.1394890T>C* | Intron2 |
| AC000158:g.1397493A>G* | Intron2 |
| AC000158:g.1398089A>G* | Intron1 |
| AC000158:g.1400792C>T* | Intron1 |
| AC000158:g.1400851_852delCT* | Intron1 |
| AC000158:g.1401172C>T* | Intron1 |
| AC000158:g.1401254G>A* | Intron1 |
| AC000158:g.1402604G>A* | Intron1 |
| AC000158:g.1404497C>T* | Intron1 |
| AC000158:g.1405957_972del16* | Intron1 |

* polymorphisms detected using next generation sequencing technique (NGS)

~ polymorphisms detected with both methods (NGS and PCR-product sequencing)
